# Microglia and neuroinflammation: An in‐depth analysis from functional diversity to disease mechanisms

**DOI:** 10.1002/ctm2.70839

**Published:** 2026-09-18

**Authors:** Jie Chen, Wangzheqi Zhang, Lizhou Song, Yan Liao, Haoling Zhang, Yiyang Xia, Derong Cui

**Affiliations:** ^1^ Department of Anesthesiology Shanghai Sixth People's Hospital Affiliated to Shanghai Jiao Tong University School of Medicine Shanghai China; ^2^ School of Anesthesiology Naval Medical University Shanghai China; ^3^ Department of Biomedical Sciences, Pusat Kanser Tun Abdullah Ahmad Badawi Universiti Sains Malaysia Kepala Batas Pulau Pinang Malaysia

**Keywords:** acute brain injury, CNS infections, microglia, neurodegenerative and demyelinating disorders, neuroinflammation, psychiatric and neurodevelopmental disorders

## Abstract

**Background:**

Microglia are essential regulators of central nervous system homeostasis and participate in immune surveillance, inflammatory regulation, phagocytosis, metabolic adaptation, and tissue repair. Recent single cell and spatial omics studies have revealed extensive microglial heterogeneity, challenging the traditional M1/M2 classification. However, the functional significance and therapeutic potential of distinct microglial states remain highly context dependent.

**Methods:**

This review summarizes recent advances in microglial biology across acute brain injury, neurodegenerative and demyelinating disorders, central nervous system infections, and psychiatric and neurodevelopmental diseases. Evidence from multiomics studies, genetic approaches, functional experiments, and emerging therapeutic strategies was integrated to evaluate mechanisms and translational challenges.

**Results:**

Microglial responses represent dynamic functional programs rather than fixed beneficial or detrimental phenotypes. Their effects on inflammation, phagocytosis, immune regulation, and tissue repair are determined by disease stage, anatomical location, and local microenvironment. Although microglia targeted therapies, including signaling modulation, depletion and repopulation, replacement strategies, and targeted delivery systems, show promise, clinical translation remains limited by insufficient human validation, model differences, and incomplete understanding of spatiotemporal regulation.

**Conclusions:**

Microglial function should be interpreted within a context dependent framework integrating cellular state, functional outcome, disease stage, and therapeutic timing. Future studies should focus on cell specific validation and human translational approaches to develop precise microglia directed therapies.

**Highlight:**

- Microglial responses are dynamic functional programs shaped by disease context, anatomical location, and temporal progression rather than fixed phenotypes.
- Single cell and spatial omics reveal extensive microglial heterogeneity, but functional validation remains essential to distinguish molecular states from biological outcomes.
- Context guided microglial modulation, including targeted delivery, immune regulation, depletion, and replacement strategies, provides new opportunities for precision therapy.

## Graphical abstract

1. Microglial functions arise from dynamic, context‐dependent programs rather than fixed M1/M2 phenotypes.

2. Inflammatory, interferon‐responsive, phagocytic/lipid‐metabolic and repair‐associated programs coexist across disease stages and brain regions.

3. Immune checkpoints, cell–cell crosstalk and cargo‐specific phagocytosis shape beneficial or detrimental outcomes.

4. Translation requires cell‐specific functional validation, disease‐stage alignment, targeted delivery and long‐term safety.

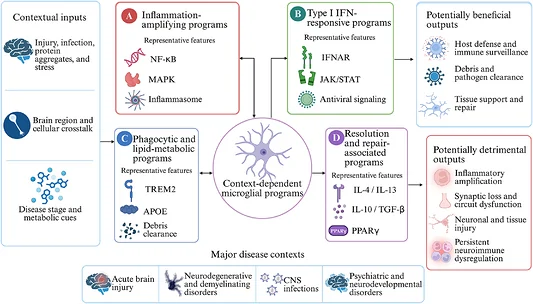

## 1 INTRODUCTION

Neuroinflammation arises in response to disturbances of central nervous system (CNS) homeostasis and is implicated in both acute brain injury and many chronic diseases of the nervous system including neurodegenerative and demyelinating disorders, CNS infections, and psychiatric and neurodevelopmental disorders. Microglia are the resident immune cells of the CNS and play several roles in environmental surveillance, inflammatory regulation, phagocytic clearance, intercellular communication and tissue repair. These functions may have a beneficial role in preserving homeostasis, yet dysregulated microglial responses can also further neurodegeneration and neuronal injury.^1^ Microglial responses to injury are thus determined by how and where the injury occurs, as well as the disease stage.

In vitro, microglial responses have traditionally been characterised using M1/M2 polarisation models; however, due to the diversity of in vivo states observed within microglia, this binary classification cannot accurately capture the breadth of potential functional states.^2^ Data from various reports demonstrate that microglia exhibit considerable disparities in transcriptional and functional characteristics based on environmental cues, anatomical locations and pathologic context.^3^ These responses are subject to regulation through proteostasis: impaired chaperone‐mediated autophagy (CMA) induces p300 and nuclear factor kappa B (NF‐κB) signalling, enhancing NOD‐like receptor protein 3 (NLRP3)‐associated inflammatory activation in microglia.^4^ Microglial responses are also heterogeneous across brain regions and stages of acute injury, as shown by single‐cell and spatial transcriptomic studies. Integrated spatial and single‐cell analysis characterised molecular changes in the peri‐infarct area where microglia and macrophages were found to be strong sources of galectins, with LGALS9‐CD44 identified as a key post‐ischemic signalling pathway through functional perturbation that connected this axis to oligodendrocyte (OL) differentiation and remyelination.^5^ In a study on the stages of perihematomal oedema (PHE) progression after intracerebral haemorrhage (ICH), single‐cell analysis of human tissue identified 12 microglial subsets and stage‐dependent SPP1 signalling mediated by Micro6, Micro1 and Micro11 while ligand receptor analysis predicted osteopontin (OPN)–CD44 interactions involving microglia in the perihematomal immune environment.^6^ In a subarachnoid haemorrhage (SAH) endovascular perforation mouse model, single‐cell profiling on day 3 revealed that SMG‐C5, SMG‐C6 and SMG‐C7 demonstrated significant SAH‐associated transcriptional alterations, together with enhanced intercellular signalling through CCL, galectin, transforming growth factor beta (TGF‐β), junction adhesion molecule (JAM), intercellular adhesion molecule (ICAM) and SPP1.^7^ The response of microglia following traumatic brain injury (TBI) has also been associated with a type I interferon (IFN)‐enriched transcriptional profile,^8^ and the expansion of S100A8‐high microglia in a porcine cardiac arrest model during the early post‐resuscitation period.^9^

These advances have fuelled efforts to target microglial function in treating neurological disease. To illustrate, a representative example is Alzheimer's disease (AD). Engagement of programmed cell death protein 1 (PD‐1) on microglia by astrocytic programmed death‐ligand 1 (PD‐L1) not only suppresses the inflammatory response to amyloid‐β (Aβ), but also promotes Aβ uptake; conversely, loss of PD‐1 signalling decreases microglial Aβ uptake and enhances amyloid deposition in experimental AD.^10^ The GLP‐1 receptor agonist NLY01 inhibited Aβ‐induced inflammatory responses in GLP‐1R‐expressing microglia and reduced microglia‐mediated reactive astrocyte formation in experimental models of AD.^11^ Spatial transcriptomic analysis confirms that enhanced microglial accumulation around amyloid plaques correlates with altered astrocytic responses and neuronal signalling in peripheral plaque microenvironments.^12^ However, these findings do not imply that the inhibition of microglial inflammatory activity should improve phagocytosis, tissue protection or neurological function. Nevertheless, the majority of the mechanistic evidence is still based on rodent models, cultured microglia or human induced pluripotent stem cell (iPSC)‐derived systems. Furthermore, cellular function or therapeutic benefit is not established by any transcriptional states alone as identified from different single‐cell or spatial analyses. Thus, the evaluation of microglial targets and responses must take into account disease stage, anatomical location, cell source and experimental model.

Next, prevention and therapeutic capabilities of microglial responses across a spectrum of acute brain injury, neurodegenerative and demyelinating disorders, CNS infections as well as psychiatric and neurodevelopmental disorders are compared to identify common and disease‐specific mechanisms. Specifically, state regulation, phagocytic role, immune checkpoints, cell‐to‐cell communication (interactions), spatiotemporal changes, depletion and replacement strategies of microglia and cellular therapies/humanised mouse models needed for human translational data are highlighted by disease stage/biology/anatomy/location with their evidence levels. Defining these common and context‐specific features might give a more solid foundation for target selection and therapeutic development.^13^

This review was designed as a general integrative narrative review rather than a systematic review or meta‐analysis.^14^ The literature searches were performed in PubMed/MEDLINE, Web of Science Core Collection and Scopus without lower date restrictions and updated until August 2026 using combinations of microglia, neuroinflammation, single‐cell omics, spatial omics with phagocytosis; immune checkpoint blockade; depletion; repopulation; replacement; neurological disorders and therapeutic delivery. Recent primary studies that provide functional or genetic evidence (cell‐specific) or single‐cell/spatial‐omics data, were emphasised where logical to establish a mechanistic basis. The review distinguished experimental context and evidence level for data derived from human tissue, patient‐derived or iPSC‐based systems,^15^ animal models and primary or immortalised microglial cultures. The goal was not to compile a comprehensive catalogue of each reported microglial alteration, but to choose representative evidence that would allow for the comparison of common and context‐dependent neuroimmune mechanisms among the broad disease categories covered in this review.

## 2 MICROGLIAL ACTIVATION, FUNCTIONAL STATE TRANSITIONS AND THERAPEUTIC MODULATION

### 2.1 From the M1/M2 framework to context‐dependent microglial states

Microglial responses are classically divided into the M1/M2 polarisation model; however, this binary classification does not accurately depict the diverse and plastic states of microglia in vivo.^16^ Hence, for the mechanistic discussion, microglial inflammatory and resolution/repair‐associated responses are treated as co‐occurring transcriptional programs and functional orientations rather than distinct or mutually exclusive in vivo subsets.

Microglial responses are dependent on their homeostatic state and the microenvironment. They rely on a specific transcriptional profile sustained by TGF‐β signals and restricted by transcription factors such as Sall1;^17^, ^18^ additionally, colony‐stimulating factor 1 receptor (CSF1R) signalling and its cytokines IL‐34 and CSF1 are crucial for postnatal development as well as homeostatic maintenance.^19^, ^20^ In this context, the local microenvironment shapes microglial responses to diverse stimuli and repair‐related signals. For example, certain cytokines (IL‐4 and IL‐13) can promote cell proliferation and self‐renewal in microglia.^21^

Toll‐like receptor (TLR) signalling elicits inflammatory‐response programs; for instance, TLR4 stimulation by lipopolysaccharide (LPS) and TLR3 stimulation by poly(I:C) resulted in distinct transcriptional and secretory responses in primary mouse microglia consistent with NF‐κB and signal transducer and activator of transcription 1 (STAT1) signalling pathways, including differential inflammatory cytokine and chemokine expression and release.^22^ In some contexts, however, inflammasome assembly functions as an amplification module by promoting caspase‐1 activation and the processing and release of IL‐1β and IL‐18. Caspase‐mediated cleavage of Gasdermin D (GSDMD) in turn induces membrane pore formation, enhancing membrane permeability and triggering downstream inflammation.^23^, ^24^, ^25^, ^26^ Multiple cytokine‐ and metabolic‐regulatory pathways lead to the emergence of these resolution‐ and repair‐associated programs. In an experimental model of ischemic stroke, the deletion of S100A9 increased anti‐inflammatory responses and efferocytosis mediated by signal transducer and activator of transcription 6 (STAT6)/peroxisome proliferator‐activated receptor gamma (PPARγ) signalling that was coupled with reduced brain injury associated with functional recovery.^27^ In addition, it is essential that these programs be viewed as functional orientations which act in concert with other response modules rather than fixed phenotypic labels.

**FIGURE 1:**
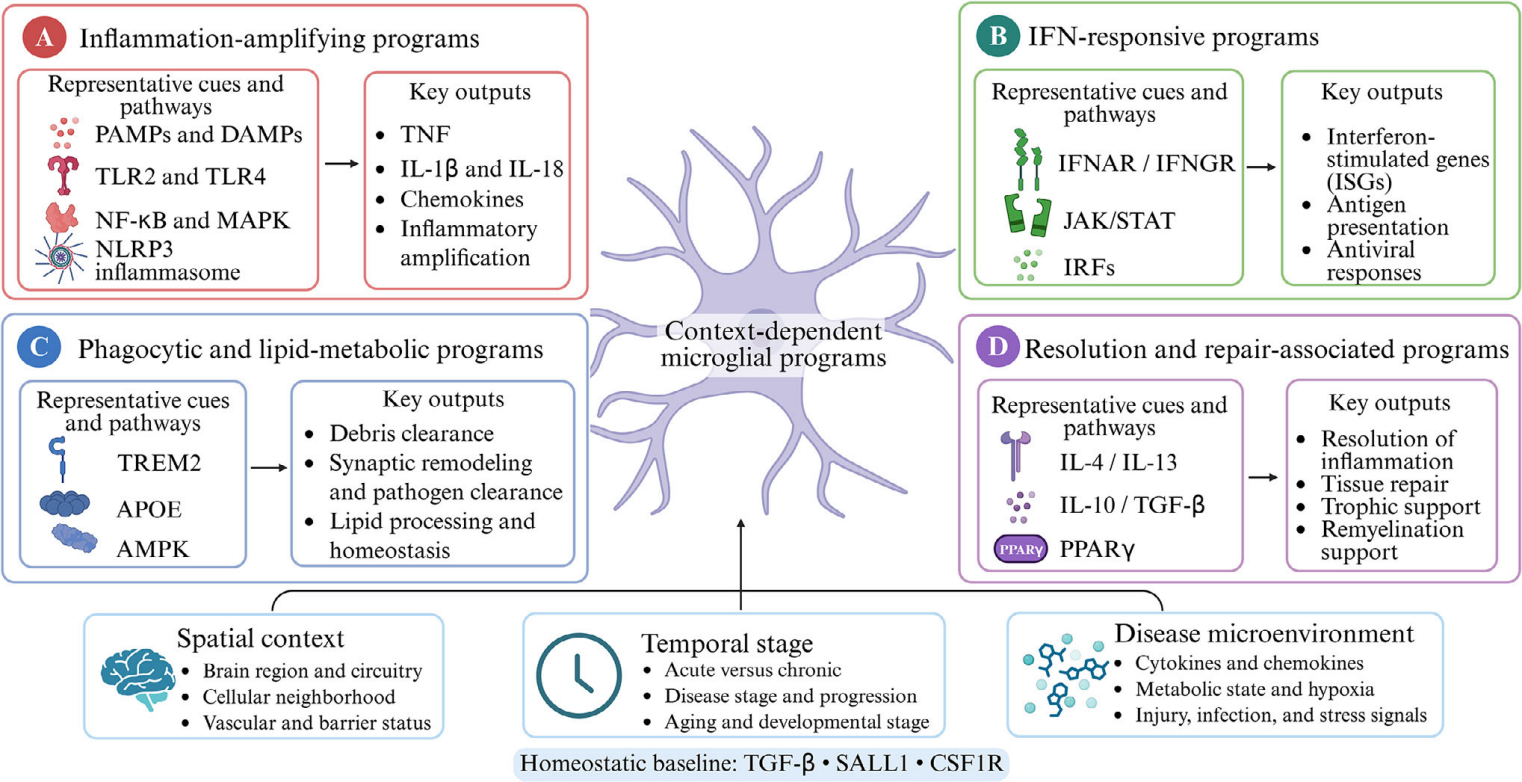
Context‐dependent functional programs of microglia during neuroinflammation. (A) Pathogen‐associated molecular pattern (PAMP)/damage‐associated molecular pattern (DAMP) sensing, TLR2/TLR4, nuclear factor kappa B (NF‐κB)/mitogen‐activated protein kinase (MAPK) and NOD‐like receptor protein 3 (NLRP3) inflammasome signalling induce tumour necrosis factor (TNF), interleukin‐1β (IL‐1β)/IL‐18, chemokine and inflammatory‐amplifying programs. (B) Interferon (IFN)‐responsive programs include protein‐coding genes regulated by interferon alpha and beta receptor (IFNAR), Janus kinase (JAK)/signal transducer and activator of transcription (STAT) and IRF signalling, and correlate with the presence of ISGs (IFN‐stimulated genes), antigen presentation and antiviral responses. (C) The phagocytic and lipid‐metabolic programs are associated with gene sets including factors such as TREM2, Apolipoprotein E (APOE) and AMP‐activated protein kinase (AMPK) for debris clearance, synapse or pathogen handling and lipid pathways, respectively. (D) Resolution‐ and repair‐associated programs include IL‐4/IL‐13, STAT6 (signal transducer and activator of transcription 6), IL‐10/TGF‐β (transforming growth factor‐beta) and PPARγ (peroxisome proliferator‐activated receptor gamma) signalling that mediate the resolution of inflammation, tissue repair and remyelination support. These programs are dynamic and influenced by the spatial context, temporal phase and local disease microenvironment, whereas TGF‐β, Sall1 and CSF1R stabilise the homeostatic set point.

Diverse and context‐dependent states of microglia are identified by single‐cell and multi‐omics studies, showing distinct coexistence, anatomical regionalism, as well as temporality across development or disease.^2^, ^3^ Microglial activation thus is better considered a heterogeneous spectrum of response programs with considerable spatiotemporal specificity. How this fits in with known mechanisms and functional outcomes is likely to be stage and microenvironment specific. Thus, the M1/M2 taxonomy is preserved in the following sections strictly when referencing terminology used in the original studies but merged into context‐dependent transcriptional and functional programs in this integrative discussion. The context‐dependence of microglial functional programs is summarised as inflammatory‐amplifying, IFN‐responsive, phagocytic/lipid‐metabolic and resolution/repair‐associated programs during neuroinflammation (Figure 1).

### 2.2 Molecular networks, genetic perturbation and causal regulation of microglial state transitions

In contrast to a unidirectionally linear polarisation pathway, state transitions of microglia derive from the integration of interacting receptor, transcriptional, metabolic, cytoskeletal, proteostatic and inflammatory networks. The contribution of these networks will depend on previous environmental exposure, genetic background, stage of disease and local microenvironment. Changes in state‐associated markers after an intervention should therefore not be regarded as evidence for effects on microglial function, which would have to be specifically assessed.^2^ Where appropriate, stronger causal evidence can be achieved using genetic deletion/knockdown and/or other pathway‐specific perturbations. Table 1 summarises representative regulatory programs, intervention strategies, functional outcomes and main evidence limitations considered throughout Section 2.

**TABLE 1:** Representative context‐dependent microglial programs and intervention strategies in neuroinflammation.

Functional program	Model/evidence	Regulator/perturbation	Key microglial response	Primary functional outcome	Main limitation	References
**A. Stimulus‐, history and response‐architecture programs**						
Stimulus‐history and genotype‐dependent inflammatory adaptation	Acute or repeated systemic LPS; PARK7/DJ‐1‐deficient mice, human iPSC‐derived microglia and region‐resolved adult mouse brain	PARK7/DJ‐1 deficiency; one, two or four low‐dose LPS exposures	PARK7 loss dampened type II IFN‐associated transcription and amoeboid remodelling; repeated LPS generated exposure‐ and region‐dependent training/tolerance signatures	The same inflammatory cue produced distinct programs according to genotype, exposure history and brain region	Single‐cell/bulk transcriptomic, morphologic and mouse evidence; links to disease progression and durable behavioural effects remain untested	^28^, ^29^
Cdk1‐dependent cytoskeletal and secretory remodelling	LPS‐, Aβ‐ or TauP301S‐fibril‐stimulated microglia; primary cultures and mouse brain‐slice/in situ analyses	Cdk1 inhibition; microtubule stabilisation and centrosomal nucleation	Cdk1 drove a centrosome‐anchored microtubule array, loss of ramification and efficient cytokine trafficking and release	Links stimulus‐induced morphology to a defined cytokine‐secretory mechanism	Primary‐cell and ex vivo/in situ evidence; sustained in vivo disease and functional outcomes were not tested	^47^
**B. Metabolic, autophagic and inflammasome control**						
Triglyceride‐ and mTORC1‐dependent lipid‐state control	Human iPSC‐derived microglia with LPS, APOE4 or APOE loss; APOE4‐humanised mouse brain slices	Triglyceride synthesis/catabolism; targeted CRISPR screen of mTORC1, lysosomal and autophagy regulators	Triglyceride‐rich lipid droplets supported inflammatory transcription, cytokine secretion and altered phagocytosis; mTORC1 regulated APOE‐dependent lipid storage	Blocking triglyceride synthesis attenuated APOE4‐associated programs and restored surveillance ex vivo	Human iPSC and ex vivo evidence plus proof‐of‐concept screening; no clinical or sustained in vivo intervention data	^30^, ^31^
CMA–p300 and NEK7 regulation of NLRP3 signalling	LPS‐stimulated BV2/primary microglia and nigral LPS mice; LPS/ATP‐stimulated BV2 cells and mouse SCI	LAMP2A‐dependent CMA; NEK7 knockdown	CMA‐mediated p300 degradation reduced p65 acetylation and NLRP3 activation, whereas NEK7 promoted NLRP3 inflammasome activation and local inflammatory signalling	CMA activation limited microglia‐mediated neuronal injury; NEK7 knockdown reduced inflammation but did not improve motor recovery	Mechanistic cell and acute mouse evidence; systemic CMA activation was not microglia‐specific, and NEK7 knockdown reduced inflammation without improving motor recovery	^4^, ^32^
**C. Immune checkpoints, population remodelling and targeted interventions**						
CD47‐dependent checkpoint control of synaptic phagocytosis	16p11.2 deletion mice; prefrontal cortex	CD47 blockade or knockdown	Increased CD47 reduced microglial synaptic engulfment; blockade or knockdown restored phagocytosis	Reduced excessive excitatory synapses and transmission and improved social novelty	Mechanistic mouse evidence; CD47 manipulation was not microglia‐specific and human efficacy was not tested	^40^
TIM‐3/TGF‐β checkpoint control of microglial homeostasis and phagocytosis	Primary microglia; microglia‐specific Havcr2 deletion; 5xFAD mice	TGF‐β–TIM‐3–SMAD2/TGFBR2 signalling; Havcr2 deletion	Havcr2 deletion increased phagocytosis and shifted microglial transcription towards pro‐phagocytic and less inflammatory programs	Reduced Aβ pathology and cognitive impairment	Cell‐targeted genetic and single‐cell mouse evidence; human therapeutic efficacy remains untested	^43^
Age‐ and injury‐dependent microglial depletion and repopulation	BLZ‐945‐treated young and aged mice; PLX5622‐mediated turnover after diffuse TBI	CSF1R inhibition followed by withdrawal	Repopulated microglia restored heterogeneity, but aged mice showed incomplete homeostatic recovery; post‐TBI turnover reduced microglial priming	Microglial density recovered; post‐TBI cognitive and depressive‐like deficits were ameliorated	Mouse evidence from different depletion paradigms; age‐ and injury‐dependent effects have not been established in humans	^51^, ^53^
Replacement of genetically dysfunctional microglia	CSF1R‐associated microgliopathy mouse models and eight patients	Bone‐marrow‐transplantation‐mediated microglial replacement	Donor‐derived cells replaced CSF1R‐deficient microglia	Reduced pathology in mice and halted disease progression during 24‐month patient follow‐up	Translational mouse and human evidence; only eight patients with limited follow‐up	^59^
Microglia‐targeted nanocarrier delivery	Microglia‐like cells; LPS/CK‐p25, hypothalamic inflammation and cancer‐cachexia mouse models	PU.1‐siRNA LNP; BBB/microglia‐targeted IRAK4‐inhibitor nanocarrier	Targeted delivery reduced PU.1 or inflammatory signalling in microglia	Reduced neuroinflammation; systemic IRAK4 inhibition also improved cachexia‐related outcomes	Preclinical cell and mouse evidence; brain‐selective LNP delivery used intracisternal administration, whereas systemic targeted delivery was evaluated only in mice	^62^, ^63^

Previous work has shown that it is not the immediate experience of a stimulus, but rather a combination of an earlier inflammatory exposure and cell‐intrinsic genetic background that conditions microglial responses. Repeated low‐dose LPS exposure in adult mice generated similar training‐ and tolerance‐associated transcriptional programs in the frontal cortex, hippocampus and striatum accompanied by microglial morphological dynamics.^28^ A different version of context dependence was produced by PARK7/DJ‐1 deficiency. At the same time, microglia from PARK7/DJ‐1‐deficient mice exhibited reduced type II IFN‐associated transcription and milder amoeboid remodelling following systemic LPS exposure, and similar transcriptional differences were found in human PARK7‐mutant iPSC‐derived microglia.^29^ Microglia contribute dynamically to CNS responses that are influenced by host exposure history and genotype.

Metabolic reprogramming controls whether microglia are able to sustain inflammatory signalling, surveillance and cargo uptake. Triglyceride‐rich lipid droplets were also revealed in human iPSC‐derived microglia stimulated with LPS, and triglyceride biosynthesis inhibition in these cells resulted in changed inflammatory transcription, cytokine secretion, phagocytosis and surveillance behaviour.^30^ A genome‐wide clustered regularly interspaced short palindromic repeats (CRISPR)‐Cas9 screen performed in iPSC‐derived microglia also revealed mTORC1, lysosomal and autophagy‐related genes as lipid storage regulators.^31^ Although this work supports a functional connection between lipid metabolism and microglial immune function, it is primarily based on engineered cellular systems in vitro that need to be validated in human brain tissue models and disease‐relevant timeframes.

Inflammatory signalling at the level of inflammasome regulation converges with proteostatic capacity. Similarly, CMA‐mediated degradation of p300 decreased NF‐κB activity and NLRP3 inflammasome activation in LPS‐stimulated microglia.^4^ NEK7 knockdown also inhibits NLRP3 activation and local inflammatory responses following spinal cord injury (SCI) but does not significantly promote motor recovery.^32^ Pro‐IL‐1β and pro‐IL‐18 are processed by downstream caspase‐1, while the subsequent cleavage of GSDMD results in pore formation on the membrane, and the release of pyroptotic mediators by its N‐terminal fragment.^33^, ^34^ The lack of functional organ recovery following NEK7 inhibition reveals that blocking a molecular endpoint of inflammation is insufficient for tissue benefit.

Approaches that employ genetic knockout, knockdown and similar perturbation strategies have been more directly amenable to tests of functional dependency of candidate pathways and assessments of the cellular specificity of their effects. Heterozygous signal transducer and activator of transcription 3 (STAT3) deletion in LysM‐expressing myeloid cells led to altered microglia/macrophage responses and aggravated infarction and neurological deficits after cerebral ischemia‐reperfusion.^35^ PARP14 knockdown and overexpression similarly linked PARP14‐dependent regulation to STAT1‐ and STAT6‐associated responses and functional recovery after SCI,^36^ suggesting a strong connection between PARP14‐dependent modulation in both cases. However, neither design fully dissociated resident microglia from infiltrating macrophages. These observations provide evidence for pathway‐specific causality within the wider myeloid compartment, but also show that genetic perturbation does not establish microglia‐specific target roles unless the manipulation provides a strong separation of resident microglia from other myeloid populations.

**FIGURE 2:**
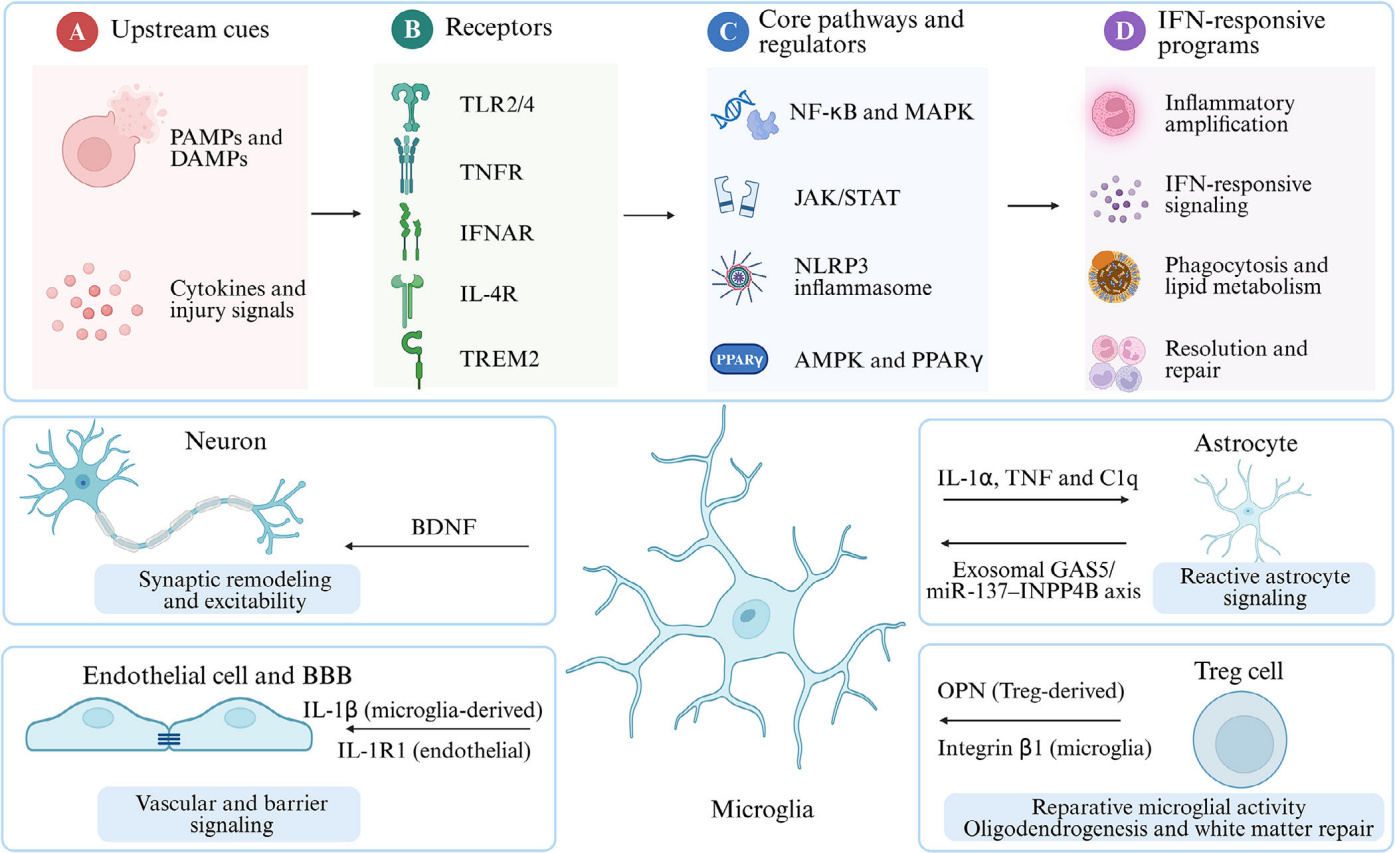
Microglial signalling networks and defined intercellular crosstalk in neuroinflammation. (A) Upstream cues include pathogen‐associated molecular patterns (PAMPs), damage‐associated molecular patterns (DAMPs), cytokines and injury signals. (B) These extracellular cues interact with receptors including TLR2/4, TNFR, interferon alpha and beta receptor (IFNAR), IL‐4R and TREM2. (C) Receptor signalling converges on core pathways and regulators including nuclear factor kappa B (NF‐κB)/mitogen‐activated protein kinase (MAPK), Janus kinase (JAK)/signal transducer and activator of transcription (STAT), the NOD‐like receptor protein 3 (NLRP3) inflammasome and AMP‐activated protein kinase (AMPK)/peroxisome proliferator‐activated receptor gamma (PPARγ). (D) These pathways regulate microglial functional programs involving inflammatory amplification, interferon (IFN) responsiveness, phagocytosis and lipid metabolism, and resolution or repair across diverse neuroinflammatory disease contexts. These outputs are then further shaped by intercellular communication. Microglial brain‐derived neurotrophic factor (BDNF) influences synaptic remodelling and neuronal excitability; microglial IL‐1α, tumour necrosis factor (TNF) and C1q promote astrocyte reactivity, while exosomal GAS5/miR‐137/INPP4B signalling feeds back to microglia from astrocytes. Microglial IL‐1 acts on endothelial IL‐1R1 to regulate vascular and barrier responses, while regulatory T cell (Treg)‐derived OPN signals through microglial integrin β1 to support reparative activity, oligodendrogenesis and white matter repair.

The collective data indicate that a multilevel regulatory model governs not only how exposure history and genotype configure the initial response architecture, but also that processing capacity is determined by metabolic and proteostatic networks, with defined inflammatory or functional outputs modulated by pathway‐specific perturbations.^37^ Importantly, these regulatory layers are not interchangeable; transcriptional state may not predict microglial function or functional consequences.^38^ Current evidence is most convincing when correlating genetic knockout or other perturbations with functional readouts and sufficiently cell‐specific experimental designs, but it continues to be limited by rodent injury models, iPSC‐derived cells, immortalised microglia, mixed microglia/macrophage attribution and short observation windows. Data from independent replication studies in humans, especially in longitudinal design and across disease stages, are still scarce. Consequently, microglial targets must be pursued only under circumstances where state assignment is in concert with cell‐specific causality, downstream function, disease context and intervention timepoint. The molecular networks and intercellular signalling pathways that regulate microglial functional programs are summarised in Figure 2.

### 2.3 Functional consequences and immune checkpoint regulation of microglial states

The microglial state transitions can change the release of inflammatory mediators, inflammasome‐associated responses, phagocytic clearance, cytoskeletal remodelling, autophagy and tissue repair. These functions can be synergistic or maladaptive, and molecular pathways shaped in cell culture models are variably protective or detrimental based on disease context, tissue environment and the type of cellular stressor.^38^, ^39^ Most of the existing evidence is based on post‐intervention markers or bulk phenotypic readouts, thus their association with particular in vivo microglial states must be regarded with caution.

Immune checkpoint and inhibitory receptor signalling represents another layer of regulation connecting microglial activation to phagocytic and inflammatory output. Microglial signal regulatory protein alpha recognises neuronal cluster of differentiation 47 (CD47) and limits synaptic engulfment. In 16p11.2 deletion mice, blockade or knockdown of CD47 signalling promoted microglial phagocytosis in the prefrontal cortex.^40^ In contrast, signalling through TREM2 promotes phagocytic activity and clearance of myelin debris and aids subsequent remyelination in demyelinating lesions.^41^ The polySia–Siglec‐E pathway also inhibits inflammatory activation, so that disruption of this pathway enhances LPS‐induced microglial responses.^42^ Other checkpoint pathways modulate inflammation or phagocytic function. Engagement of microglial PD‐1 by astrocytic PD‐L1 dampens inflammatory cytokine production, but also promotes Aβ uptake,^10^ and microglial T‐cell immunoglobulin and mucin‐domain containing‐3 (TIM‐3) supports homeostasis by interacting with the TGF‐β signalling pathway; genetic deletion of TIM‐3 activates phagocytic activity and influences disease‐related transcriptional programs.^43^ LAG‐3 acts as a further inhibitory mechanism, with IFN‐γ/STAT1‐dependent LAG‐3 expression and increased nitric oxide production following LAG‐3 knockdown in activated microglia.^44^ Collectively, these results suggest that checkpoint regulation is not universally protective or inhibitory but instead its functional outcome is specific to the receptor, cellular ligand source, phagocytic cargo and the disease context and timing of the microglial response.

An example of how different molecular programs can modulate specific functional arms of the microglial response is in an acute ischemic injury system. Tat‐NTS promotes the SUMOylation of Annexin A1, which inhibits NF‐κB‐mediated inflammatory signalling in microglia and correlates with reduced infarct volume and enhanced neurological outcomes following ischemia‐reperfusion.^45^ Recent findings in tMCAO and oxygen‐glucose deprivation/reoxygenation (OGD/R) models show that Aloe‐emodin protects microglia against ischemia‐induced injury by suppressing NLRP3 inflammasome activation and pyroptosis.^46^ Combined, these findings suggest that microglia‐mediated signalling does not represent a general directional state transition but rather varies in a context‐ and process‐dependent manner across multiple injury stages.

Checkpoint and receptor signals dictate cargo recognition, while cytoskeletal and proteostatic mechanisms regulate the execution of a microglial response. Cdk1‐dependent microtubule remodelling aided stimulus‐induced morphological alterations and cytokine trafficking following treatment with LPS, Aβ or TauP301S fibrils in primary cultures, and mouse brain‐slice or in situ analyses.^47^ Although this study did not detect sustained in vivo disease or behavioural outcomes, metformin increased microglial autophagic flux and myelin debris clearance in SCI models, and the effects were attenuated by inhibition of autophagy.^48^ These results link receptor signalling with cytoskeletal remodelling, autophagic processing and cargo clearance while refraining from assigning these alterations to any stable microglial phenotype.

Together, the immune checkpoints, cytoskeletal remodelling, lipid‐metabolic and lysosomal capacity, inflammasome activity and autophagic processing collectively regulate distinct steps of target recognition, engulfment, cargo processing and inflammatory output. Moreover, higher levels of phagocytosis cannot be assumed to be beneficial, as the functional outcome depends on whether the cargo is pathological material, protein aggregates, myelin debris or intact synaptic structures,^49^ and may be further influenced by whether intracellular processing capacity is sufficient to finish clearance. The most compelling data are provided when genetic deletion or pathway inhibition affects a defined functional cellular phenotype, but many studies are still based on rodent injury models, cultured microglia or pharmacological interventions with sparse verification in human tissue. Overall, checkpoint expression or state‐associated markers do not define therapeutic benefit unless linked to cell‐specific causality (i.e., associated with functional outcome and disease‐stage alignment) while preserving the essential clearance functions required for curative potential.

### 2.4 Microglial depletion, repopulation and replacement

Microglial depletion, repopulation and replacement modulate the balance of different cell types within the microglial compartment, not one particular inflammatory arm, and as a result provide orthogonal approaches to examine microglial function. Depletion is the process of removing resident microglia, repopulation uses endogenous cells to replenish the depleted compartment and replacement fills in the available niche with exogenous or donor‐derived cells.^50^ Such strategies differ in cell source (bone marrow vs. peripheral blood), timing of transplantation, persistence as well as the degree to which pre‐existing microglial programs are eliminated or reinstated (the latter primarily generating injury‐induced/developmentally programmed myeloid cells). Their effects should therefore be viewed depending on whether the intervention depletes, replenishes or replaces resident microglia.

CSF1R signalling is crucial for supporting microglial survival and maintenance and strongly influences both endogenous depletion and repopulation. Pharmacological inhibition of CSF1R markedly reduces resident microglial numbers, while withdrawal of the inhibitor allows for rapid restoration of the depleted niche. Microglia in mice treated with the CSF1R inhibitor BLZ‐945 repopulated by 7 days post‐treatment.^51^ It is important to note that numerical recovery does not equal functional normalisation. Single‐cell analysis revealed that repopulated microglia reformed the major functional clusters but with continued upregulation of pro‐inflammatory genes, while aging further exacerbated replicating and non‐classical inflammatory lineages in addition to hindering restoration of homeostatic phenotype.^51^ Depletion and subsequent renewal normalised sensitised pro‐inflammatory signalling in organotypic brain‐slice cultures,^52^ while accelerated microglial turnover ameliorated chronic cortical inflammation, pathological immune reactivity and persistent cognitive impairment after diffuse brain injury.^53^ Therefore, repopulation can reorganise a dysregulated microglial compartment, but cell number restoration alone should not be interpreted as a full return to the pre‐injury functional state.

Microglial depletion also has strongly disease‐dependent consequences, as microglia removal can simultaneously extinguish inflammatory actions that may be detrimental while simultaneously removing protective functions that are critical for maintaining homeostasis. In an ex vivo West Nile virus infection model, depletion of microglia led to increased viral titres and cell death, highlighting the important role played by resident microglia in the antiviral and tissue‐protective responses.^54^ Likewise, depletion of microglia hastened prion disease pathogenesis without demonstrating an increase in prion burden in the brain.^55^ These results are opposite the positive effects of microglial turnover during chronic post‐injury inflammation and show that depletion should not be considered a broadly anti‐inflammatory or neuroprotective treatment. Rather, its effects depend on the predominance of specific microglial functions at the time of intervention, whether those are pathogen restriction, phagocytic clearance, inflammatory amplification or tissue support.

The approaches above extend to exogenous microglial replacement, where donor‐derived cells with intact or genetically corrected functions are introduced into the available niche. Under a CNS‐wide replacement paradigm, however, haematopoietic stem cells (but not immediate myeloid or monocyte progenitors) exhibited whole bone marrow‐equivalent engraftment capacity and donor‐derived cells ameliorated neurodegeneration, motor and balance deficits, and increased survival in Prosaposin‐deficient mice.^56^ Microglial progenitors derived from human iPSCs restored a homeostatic microglial signature and prevented major neuropathological features in a chimeric model of CSF1R‐related leukoencephalopathy, while transplantation of CRISPR‐corrected patient‐derived cells reduced pre‐existing pathological abnormalities.^57^ Sca1‐negative committed progenitor cells achieved efficient replacement under brain‐restricted conditioning without conventional systemic myeloablative preconditioning and demonstrated improved disease manifestations in the Sandhoff disease mouse model, while ubiquitous engraftment potential was shown by human embryonic stem cell‐derived myeloid progenitors.^58^ Clinical evidence has begun to emerge in CSF1R‐associated microgliopathy, where it was found that bone marrow transplantation could replace CSF1R‐deficient microglia in eight patients and halt disease progression over a follow‐up of 24 months.^59^

However, they should not be seen as interchangeable or equally beneficial strategies; there are also downsides to depletion, repopulation and replacement. Small‐molecule CSF1R inhibition is not microglia selective, with PLX5622 impacting both peripheral myeloid and lymphoid compartments and achieving persistent macrophage changes after treatment termination.^60^ Consequently, phenotypes observed following CSF1R inhibition cannot always be explained by depletion of resident microglia. Replacement carries more uncertainties that concern the origins of donor cells, conditioning and engraftment efficiency, regional distribution and immune compatibility with long‐term acquisition of resident microglial identity. Although replacement in primary microgliopathies has greater preclinical and early human support, extrapolation to those with secondary microglial dysfunction is currently premature. Translation will depend on selecting an appropriate strategy for the disease and window for intervention, along with demonstrating that repopulated or replacement cells achieve necessary functions without creating additional immune dysregulation.

### 2.5 Emerging microglia‐directed therapeutic and delivery strategies

Importantly, the context dependence and functional diversity of microglial responses are a challenge to translation for therapeutic development. A generalised inhibition of microglial activation may have decreased deleterious inflammatory signalling but could work against surveillance, phagocytic clearance, pathogen defence and tissue repair. Thus, emerging strategies increasingly emphasise precisely controlling the site/time/context of action for a therapeutic. In this review, current strategies include nanoparticle‐mediated delivery of nucleic acids or small‐molecule inhibitors, local or biomaterial‐assisted administration and non‐pharmacological modulation through photobiomodulation. Their main theoretical benefit may not only be due to changing inflammatory output but also improving target‐cell access, diminishing off‐target distribution or providing spatial and temporal control of microglial modulation.^61^

Delivery of nanoparticles represents a direct approach to increase therapeutic exposure to microglia: this strategy deals with barriers both to intracellular nucleic‐acid delivery and CNS access. A dual‐carrier system incorporates several delivery tactics into one microglia‐oriented lipid nanoparticle formulation that enables preferential delivery to human stem cell‐derived microglia‐like cells and inflammatory cells via different uptake pathways, while intracisternal administration increases brain‐selective expression compared with systemic administration. Delivery of PU.1‐targeting siRNA reduced PU.1 expression and reduced neuroinflammation in LPS‐treated and CK‐p25 mice.^62^ Utilising a complementary systemic approach, the IRAK4 inhibitor zimlovisertib was delivered via a blood‐brain barrier (BBB)‐penetrating peptide combined with a microglia‐binding peptide. After intravenous administration, the dual‐functionalised nanocarrier FIGS‐tT‐MWNTs accumulated in both brain and hypothalamus, demonstrated specific recognition with IBA1‐positive microglia, and suppressed hypothalamic inflammatory mediators specifically in models of acute hypothalamic inflammation and cancer‐cachexia‐associated neuroinflammation.^63^ Microglial state may itself depend on the performance of lipid nanoparticles. A hyaluronic‐acid‐modified formulation, from a screening of 216 formulations, was found to successfully deliver IL10 mRNA to LPS‐activated BV2 cells and human iPSC‐derived microglia that both expressed CD44 for increased IL‐10 while reducing the tumour necrosis factor (TNF)‐α‐mediated inflammatory response.^64^ Collectively, these studies demonstrate that therapeutic delivery can be tuned at several levels: route of administration, capability to cross the BBB, CD11b+ cell targeting, cargo selection and the state‐of‐response of microglia within the recipient animal.

Alternative delivery and biomaterial‐assisted approaches provide additional avenues whereby therapeutic exposure can be modulated without necessarily being considered microglial‐specific. In an AD mouse model, intranasal cardiolipin liposomes encapsulating carrier‐free curcumin nanoparticles showed the ability to respond to this oxidative microenvironment and to inhibit Aβ aggregation as well as induce microglial Aβ uptake and clearance in a TLR4/NF‐κB‐associated inflammatory signalling‐dependent manner; these biological effects translated into reduced Aβ burden, neuroinflammation and cognitive deficits.^65^ While the original study viewed these alterations through an M1 to M2 polarisation lens, measures of the secreted Aβ and inflammatory changes are better understood as coordinated changes in microglial Aβ processing, phagocytic clearance and inflammatory signalling. Experimental TBI specifically evaluated induced neural stem cell‐derived exosomes that were delivered either locally in an injectable hydrogel or systemically through RVG targeting. Both approaches enhanced neurological outcomes, and single‐cell analysis showed that microglia were a key population responsive to treatment and subsets of specific microglial subpopulations altered by treatment predicted neuron–microglia signalling.^66^ An independent TA/QCS injectable hydrogel has reduced neuronal ferroptosis in addition to decreased microglial and astrocytic activation and improved neurological recovery 7 days after TBI.^67^ Notably, this latter finding demonstrated that localised delivery of the biomaterial is able to alter the inflammatory tissue milieu in the absence of establishing a microglia‐specific modality.

Physical modulation represents a mechanistically different approach whereby microglial responses are altered in the absence of traditional pharmacological delivery. 4 J/cm^2^ photobiomodulation at 1070 nm (10 Hz) changed microglial activation and increased miR‐7670‐3p levels in microglia‐derived exosomes in the brains of 5xFAD mice. This miRNA inhibited ATF6‐mediated ER stress, reduced inflammatory responses, maintained the integrity of dendritic spines and correlated with improved cognitive performance.^68^ In a mild TBI rat model, transcutaneous photobiomodulation at 660, 810 or combined 660/810 nm resulted in improved behavioural recovery, with the strongest overall effect noted for 810 nm. At 3 days after injury, treatment also decreased CD11b‐positive microglial and GFAP‐positive astrocytic activation in conjunction with cleaved caspase‐3.^69^ These investigations span microglial modulation beyond the aspect of drug delivery but also illustrate that photobiomodulation should never be regarded as a single mechanism, as factors including wavelength, energy delivery, timing of treatment, depth of penetration and disease tissue context can account for observed biological responses.

Taken together, these exciting strategies provide a comprehensive evolution of microglia‐targeted therapy away from classical indiscriminate suppression towards more effective control over therapeutic cargo, cellular targeting, tissue localisation and timing of intervention. However, the degree of target specificity varies widely across strategies. This includes peptide‐functionalised nanocarriers, which can be used to validate preferential uptake by microglial cells.^63^ In contrast, a significant number of intranasal formulations, injectable hydrogels, exosome‐based treatments and photobiomodulation frequently impact multiple neural and glial populations. Most efficacy and mechanistic evidence also remains preclinical, relying on rodent disease models, BV2 cells, human iPSC‐derived microglia or short observation periods. More broadly, cross‐study comparison of therapeutic effect magnitude is hampered by (i) differences in dose, route of administration, formulation, treatment schedule and disease model used; and (ii) the lack of independent replication and human clinical validation for many of these strategies. Although intracisternal or local administration improves CNS exposure, these routes are invasive. In contrast, systemic delivery must overcome barriers related to BBB penetration, peripheral biodistribution and off‐target uptake.^63^ Then photobiomodulation becomes further dependent on wavelength, dose, penetration depth and treatment schedule. Thus, future microglia‐directed approaches should validate these five essential components of therapies: cellular specificity, delivery efficiency, concordance with disease stage and functional outcome as well as long‐term safety jointly rather than pre‐assuming the therapeutic benefit of a reduced inflammatory marker for its secretion in a particular cell type.

## 3 MULTIFUNCTIONALITY OF MICROGLIA IN NEUROINFLAMMATION

### 3.1 Immune surveillance and neuroprotection

Microglia continuously survey the CNS parenchyma through highly motile processes under homeostatic conditions.^70^ This regulation is also constitutive surveillance, which enables them to discern minute fluctuations in extracellular signalling, local cellular homeostasis and tissue integrity prior to overt structural damage. Thus, homeostatic surveillance does not represent a passive resting state but instead constitutes the functional baseline from which directed responses to local challenges are initiated. These initial sensing roles can swiftly transition to chemotactic recruitment, lesion containment, phagocytic disposal and other tissue‐supportive functions upon damage of pernicious nature.

Rapid extension of microglial processes towards sites of tissue injury is an early functional response of microglial surveillance. Following focal injury, extracellular ATP released from damaged tissue rapidly directs microglial process extension towards the lesion before significant soma migration is evident.^71^ It can be a spatially restricted interface between an injurious stimulus affecting the tissue and parenchyma that may partition containment and clearance responses thereafter. Recruitment occurring prior to more extensive morphological and transcriptional remodelling suggests that early microglial responses consist of rapid sensing and process motility, before functional programs are established.

In addition to their recruitment, which can occur rapidly, microglia also coordinate a variety of response phases including target recognition, phagocytic clearance and supportive functions. Phagocytic capacity is plastic and is moulded by the microenvironment in which it operates. The opposing effects of IFN‐γ, IL‐4/IL‐13 and GM‐CSF on Fcγ receptor expression and IgG‐mediated phagocytosis,^72^ in this study illustrate that the cytokine context can dictate how microglia recognise and clear opsonised targets. Importantly, the impact of this process is dictated by microglial activation state, receptor repertoire, cargo identity and downstream effector pathways. In this way, phagocytosis can facilitate clearance of damaged debris and promote tissue regeneration, yet inappropriate target recognition or excessive engulfment by other cells may redirect the same functional apparatus into one that contributes to tissue damage.

However, these functions are still context‐dependent and should not be considered inherent features of a static microglial state.^73^ Microglial activation is a complex and finely regulated event in which rapid recruitment and appropriately targeted clearance may serve to limit tissue injury, whereas prolonged inflammatory signalling, excessive phagocytic burden or engulfment of viable cellular and synaptic elements can become pathological. Therefore, the same functional process may result in different outcomes based on localisation of the lesion, stage of disease progression, localised cytokine and metabolic signals to macrophages as well as identity of the material being cleared. Thus, immune surveillance and neuroprotection are better characterised as context‐dependent functional outputs constituted from coordinated sensing, migration, recognition and clearance rather than properties of a single beneficial microglial phenotype.

### 3.2 Neuronal injury and exacerbation of inflammation

Neuronal injury accompanied by microglial activation is achieved more through the convergence of multiple effector processes than as the result of one limited inflammatory state.^74^, ^75^ Inflammasome signalling, oxidative stress, autophagy‐related responses and microglia‐derived inflammatory mediators have been implicated in promoting tissue injury across models of neurodegeneration, trauma, demyelination and ischemic‐like insults.^76^ Depending upon the initiating insult, duration of activation and cellular context, these pathways variably contribute, as specific damaging processes will need to be disentangled from the overall transcriptional state in which they occur.

One pathway for the conversion of microglial sensing into inflammatory amplification and tissue injury is through inflammasome signalling. TMEM16F knockdown decreased NLRP3‐related inflammatory signalling and pro‐inflammatory cytokine production in APP/PS1 mice and Aβ‐induced human microglia while also resulting in reduced apoptosis and Aβ buildup.^77^ In traumatic SCI models, pharmacological mTOR inhibition with rapamycin before injury suppressed AIM2/ASC/caspase‐1‐associated signalling and was associated with improved neuronal and tissue outcomes.^78^ While the design‐based evidence differs, as pharmacological pretreatment supports mTOR/AIM2 pathway involvement and genetic knockdown supports TMEM16F participation, collectively these studies suggest that distinct inflammasome pathways may mediate neuronal injury during both chronic neurodegeneration and acute trauma.

Oxidative stress and autophagic responses represent a second mechanism connecting the activation of microglial cells with damage of the surrounding tissue. Hv1 deficiency attenuated microglial reactive oxygen species (ROS) production and autophagic activity, which corresponded with reduced disease severity in the lysophosphatidylcholine (LPC) demyelination model, thus linking redox signalling to autophagy‐associated injury.^79^ Salvianolate inhibited oxygen‐glucose deprivation (OGD)‐induced ROS production, TLR4 signalling and expression of IL‐6 and TNF‐α in microglia, while conditioned‐medium experiments indicated significantly reduced neuronal apoptosis after suppression of the microglial inflammatory response.^80^ These results are consistent with a potential mechanism for microglia‐neuron crosstalk in which pathological oxidative and inflammatory outputs from OGD‐exposed microglia could exert downstream effects on neighbouring neurons. In addition, they suggest that autophagy‐related responses must not be taken to universally protect against injury as their functional consequences are conditioned by the context of injury and the upstream signals with which they are coupled.

**FIGURE 3:**
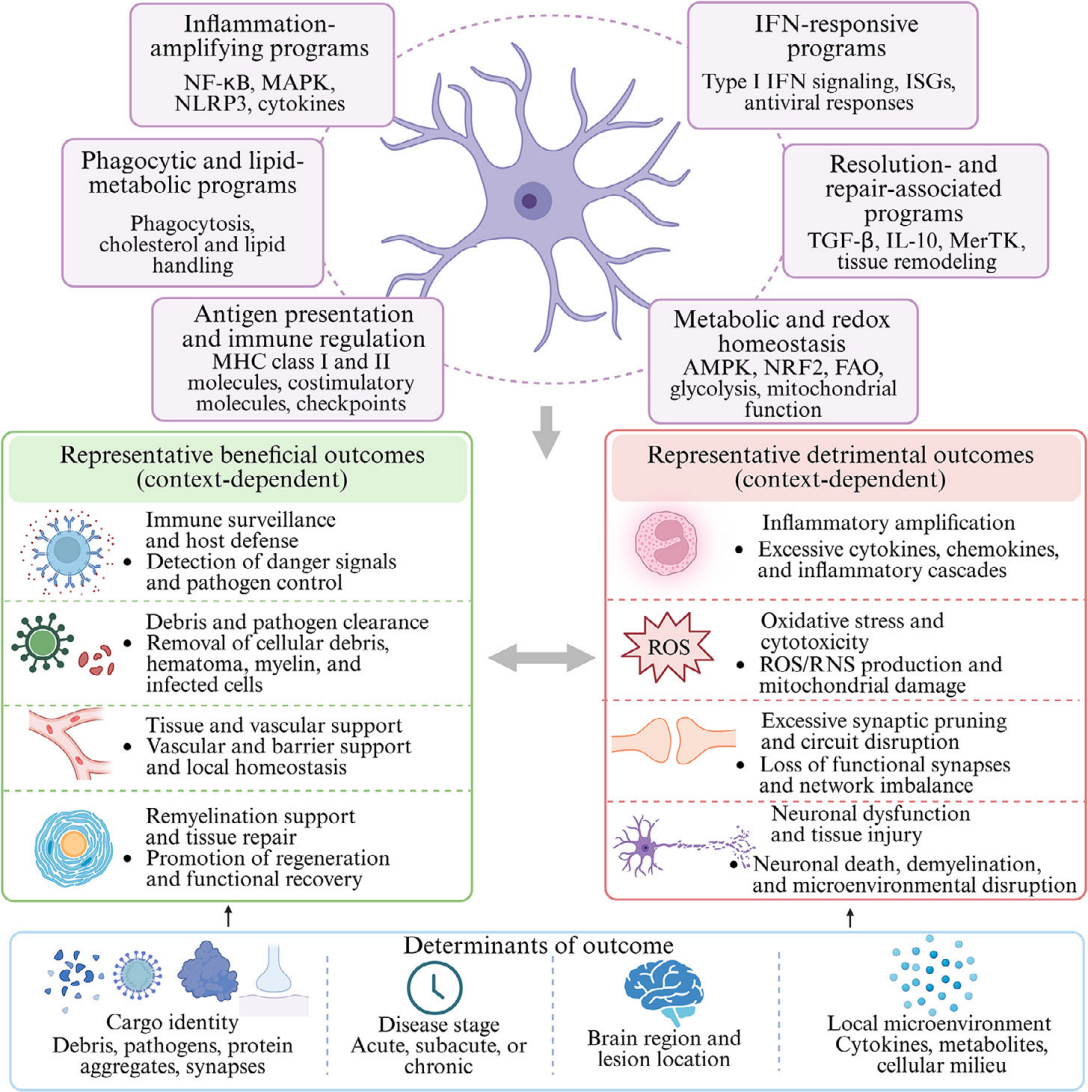
Context‐dependent functional consequences of microglial programs in neuroinflammation. Microglial responses consist of overlapping inflammatory‐amplifying, interferon (IFN)‐responsive, phagocytic/lipid‐metabolic, resolution/repair‐associated, antigen‐presentation/immune‐regulatory and metabolic/redox programs. They are not inherently beneficial or detrimental in their function. Examples of positive effects include immune monitoring and host protection, clearance of debris and pathogens, support for vessels and tissues, and net effects on remyelination or tissue repair. Examples of damaging effects consist of inflammatory amplification, oxidative stress and cytotoxicity, excessive synaptic pruning as well as circuit dysfunction and neuronal or tissue injury. Whether these effects are in the same or different directions is dictated by cargo identity, disease stage, brain region or lesion location and the local microenvironment. Hence, such microglial programs can lead to varied phenotypes in divergent biological environments.

Crucially, model systems exhibiting higher levels of microglial inflammatory activation do not always correlate with greater tissue damage. In an experimental model of multiple sclerosis (MS), EAE in LEWzizi rats with pre‐existing microglial activation and neurodegeneration redistributed the EAE‐associated neuroinflammation into forebrain regions but did not aggravate tissue injury overall.^81^ Thus, this paradox is a strong argument against considering isolated changes in inflammatory markers as conclusive evidence of neuroprotection or neurotoxicity. The majority of mechanistic evidence presented in this section is still based on rodent disease models or cultured microglia, and it is not known to what extent these pathways accurately reflect human atrophy stages, regional microenvironments and cell‐specific functions. Figure 3 summarises the contextual beneficial and detrimental effects of overlapping microglial functional programs.

### 3.3 Microglial crosstalk with neural, glial, vascular and immune cells

Microglial functions depend on signalling both cell‐intrinsically and via reciprocal communication with neurons, astrocytes, vascular cells and infiltrating immune populations. Such interactions can modify neuronal excitability, glial reactivity, vascular inflammatory signalling, phagocytic activities and tissue repair as well. Such cross‐talk may be characterised mechanistically in terms of the signal‐producing cell, as well as some mediator (or receptor pair), a responding cell and the functional consequence.^82^ Since the impact of these interactions depends upon disease context and stage, intercellular signalling is a major determinant in how microglial responses become transduced into tissue injury or recovery.

Neuronal activity and circuits can be directly altered by signalling from microglia to neurons. This study found that, after peripheral nerve injury in male mice, microglia appeared ramified and at normal density with increased brain‐derived neurotrophic factor (BDNF) expression within the primary somatosensory cortex.^83^ Conditional deletion of BDNF from microglia suppressed synaptic remodelling and pyramidal hyperactivation, also protecting against mechanical allodynia, whereas S1‐targeted depletion of BDNF from microglia produced similar protective effects.^83^ Together, these findings provide a functional connection between microglial BDNF and neuronal circuit remodelling and show that robust microglia‐to‐neuron signalling can occur independent from classical morphological activation of the cell. Nevertheless, the long‐term BDNF effects and the key causal experiments were not verified in females so that generalisation across sexes is still difficult.

Microglia and astrocytes form a bidirectional signalling network initialised by microglia, which may amplify or modulate local inflammation. In experimental neuroinflammation, IL‐1α, TNF and C1q were collectively required and sufficient for LPS‐activated microglia to induce a gene expression program in astrocytes that fully supports the transition into a neurotoxic reactive phenotype associated with loss of synaptic‐support and neuronal‐survival programs.^84^ In the chronic postsurgical pain rat model, changes in astrocyte reactivity occurred after microglial activation; furthermore, microglial inhibition and manipulation of CXCR7/PI3K/Akt signalling altered C3‐ and S100A10‐associated astrocytic responses along with mechanical allodynia.^85^ Signalling may also occur from astrocytes to microglia. Astrocyte‐derived exosomal signalling through the GAS5/miR‐137/INPP4B axis via regulation of PI3K/Akt activity modulated microglial apoptosis and inflammatory responses in a cardiac arrest/cardiopulmonary resuscitation model under OGD/R conditions.^86^ These studies, together, suggest that microglia–astrocyte communication is reciprocal and its functional consequence depends on the initiating cellular stress and the molecular signal.^87^

The communication of microglial cells with vascular and peripheral immune cells additionally ties local CNS responses to the systemic effects of inflammation and tissue repair.^88^ In the spinal cord, repeated systemic LPS exposure augmented microglial IL‐1 production and also enhanced communication with endothelial cells in an IL‐1R1‐dependent manner; using cell‐type‐specific approaches to silence IL‐1R1 demonstrated that this cross‐talk was required for local pro‐inflammatory microglial response and protection against metachronous ischemic spinal motor neuron loss.^89^ In the chronic recovery phase following experimental ischemic stroke, brain‐infiltrating regulatory T cells (Tregs) mediated repair through induction of reparative microglial activity, which was at least partially dependent on OPN–integrin β1 signalling.^90^ Treg depletion inhibited oligodendrogenesis and white matter repair, while microglia depletion diminished the promoting effects of exogenous Treg cells; loss of Treg‐derived OPN or integrin β1 signalling in C‐X3‐C motif chemokine receptor 1 (Cx3cr1)‐expressing cells further decreased Treg‐enhanced oligodendrogenesis.^90^ Vascular and peripheral immune partners can selectively divert microglial functions utilising a series of defined signalling pathways rather than by global inflammatory activation.

**FIGURE 4:**
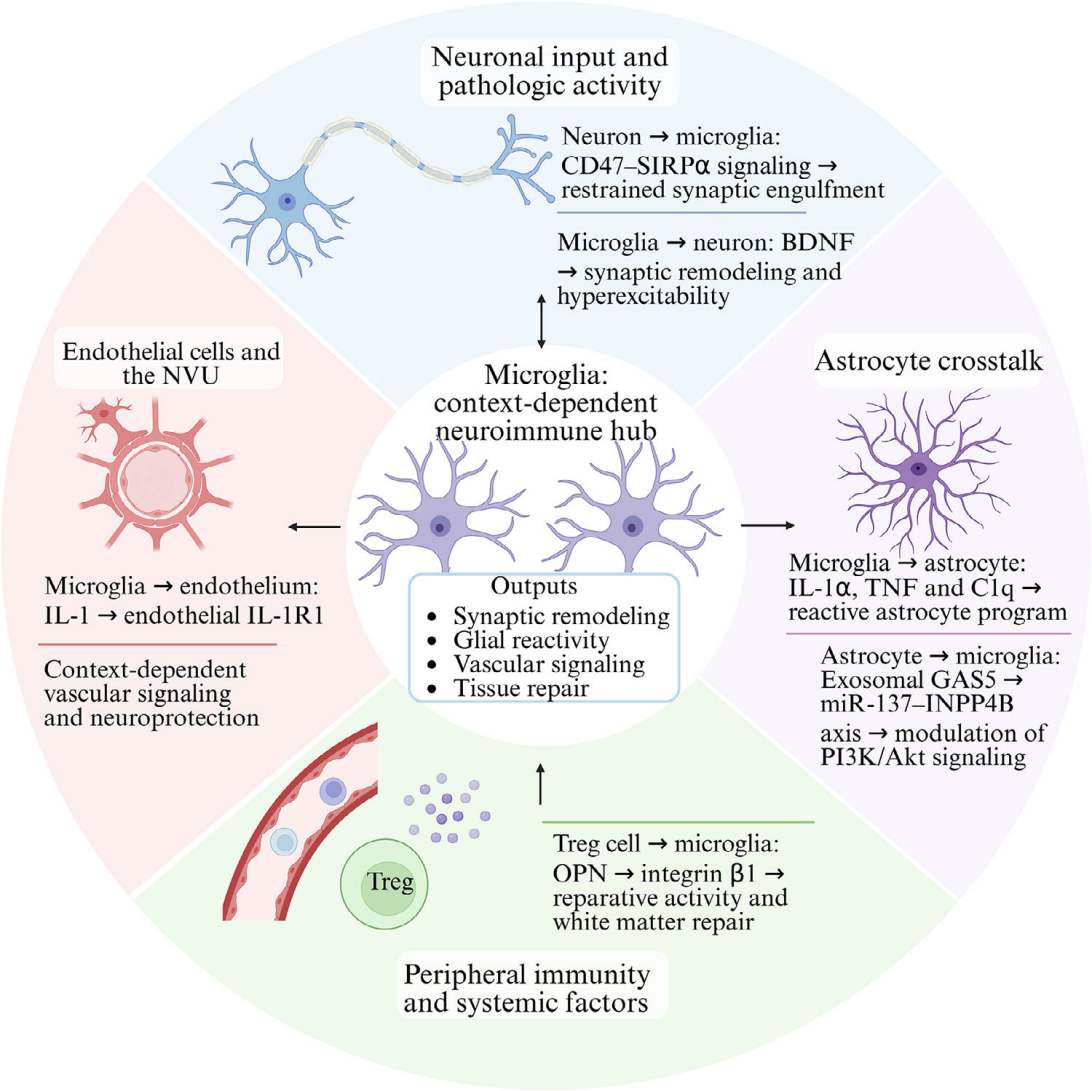
Microglia as a context‐dependent hub of neuroimmune intercellular communication. Microglia integrate signals from neurons, astrocytes, endothelial cells and peripheral immune populations to turn them into context‐dependent neuroimmune outputs. Neuronal cluster of differentiation 47 (CD47) may inhibit microglial synaptic engulfment, while microglial BDNF can facilitate neuronal synaptic remodelling and hyperexcitability. In addition to the above, microglial IL‐1α, tumour necrosis factor (TNF) and C1q activate programs for reactive astrocytes; conversely, signalling from astrocyte‐derived exosomal GAS5/miR‐137/INPP4B alters responses in microglia associated with PI3K/Akt. Microglial IL‐1 signalling through endothelial IL‐1R1 differentially modulates context‐dependent vascular responses while infiltrating regulatory T cell (Treg) cells drive reparative microglial activity via OPN‐integrin β1 signalling. Through these interactions, microglia regulate synaptic remodelling, glial reactivity, vascular signalling and tissue repair, highlighting the fact that intercellular communication can also yield divergent functional outcomes depending on disease and/or tissue context.

Taken together, these examples illustrate that microglial crosstalk cannot simply be conceptualised as an inflammatory or protective network absent context. Microglial BDNF induces neuronal hyperexcitability and maladaptive circuit remodelling.^91^ IL‐1 dependence cannot always be determined by these approaches and in a different experimental context, IL‐1‐dependent interaction with the endothelial cell can mediate neuroprotection. Meanwhile, cytokines secreted by microglia can initiate astrocytic responses that may mediate neurotoxicity whereas Treg cell infiltration into the CNS in response to such signals can modify and redirect microglial activity towards white matter repair. Crucially, the strength and breadth of these conclusions are study‐dependent, ranging from cell‐type/region‐focused genetic perturbation to pharmacological intervention, coculture systems and transcriptomic inference. Mechanisms also need to be thoroughly interpreted in light of species, sex, anatomical region, disease stage and interacting cell population before extrapolating findings directly to human disease. This has led to the impression that intercellular communication is a context‐dependent regulator of microglial output, rather than evidence for a fixed beneficial or detrimental microglial state.^82^ Figure 4 summarises those context‐dependent neuroimmune interactions and their main functional outputs.

### 3.4 Spatiotemporal heterogeneity of microglia in acute brain injury and therapeutic windows

As lesions can often be reproduced in a more well‐defined spatiotemporal fashion than other disease processes, acute brain injury presents a relatively tractable space for studying microglial heterogeneity. Indeed, microglial responses with respect to anatomical area, lesion compartment, cellular composition and disease stage do not follow a uniform temporal pattern.^92^ The pattern of the precipitating injury may also continue to influence these responses. Thus, in line with this perspective, acute microglial responses are considered in terms of spatial, temporal and injury specificity among individual studies, and specific focus is placed on the degree to which transcriptomic differences are corroborated by experimental demonstration of established cellular functions.

Spatial heterogeneity is especially prominent following ischemic stroke. Single‐cell and spatial transcriptomic analyses of MCAO mice across 3, 12 and 72 h revealed two populations: ICAM, which exhibited robust glycolytic and inflammatory programs, and ischemic penumbra‐associated microglia, which were more moderate in their inflammation level but also showed increased tricarboxylic acid cycle, oxidative phosphorylation (OXPHOS) activity, and myelin‐supportive features.^93^ In a distinct mouse study at 3 days following cerebral ischemia, proliferative MKI67+ microglia co‐identified with CH25H+ and OASL+ ischemic stroke‐associated subclusters; CH25H+ microglia displayed enhanced phagocytic features as well as increased infarct volume when Ch25h was deficient, while OASL+ microglia were strongly linked to type I IFN signalling and were more prevalent in aged ischemic brains.^94^ These results suggest that heterogeneity of microglia subsequent to focal ischemia is not only gauged by lesion geography, but also by differences in metabolic activity, phagocytic function and inflammatory signalling consistent with the age of the host.

Temporal heterogeneity imposes a second layer of complexity in ischemic microglial responses. Single‐cell analysis of a rat cerebral ischemia‐reperfusion model revealed seven microglial subclusters alongside enhanced inflammatory signalling, glycolysis, lipid metabolism and decreased TCA cycle and OXPHOS activity; regulatory analyses further connected ATF3 to CH25H‐ and SPP1‐related programs, as well as showed that ATF3 overexpression augmented CH25H expression and cytokine secretion.^95^ Direct evidence supporting stage‐dependent functional effects comes from STAT1 deletion targeted to Mi/MΦ and showing no reduction in the size of neuronal death 24 h after MCAO but attenuation of subsequent inflammatory responses, reduced long‐term white matter injury (within weeks) and improved functional recovery.^96^ These studies also highlight a key distinction: single‐cell profiling is useful to identify candidate regulatory programs, but must be followed by temporally resolved perturbation to reveal when those candidate programs exert material influences on tissue outcome.

Haemorrhagic brain injury exhibits further spatiotemporal and intercellular dynamics. In single‐cell RNA sequencing (scRNA‐seq) of PHE tissue from nine patients with basal ganglia ICH, 12 microglial subclusters were evident across samples taken in 0–6 h, 6–24 h and 24–48 h after haemorrhage, and major contributions to intercommunication among these microglial subclusters were related to SPP1‐associated signalling and predicted OPN–CD44 interactions within the evolving perihematomal immune environment.^6^ In a mouse perforation model of SAH, microglial accumulation at the site of injury developed from day 1 to day 10. A continuum of 10 microglial clusters was identified by single‐cell analysis on day 3, and CCL, galectins, TGF‐β, adhesion‐related and SPP1‐associated signalling networks were involved in post‐SAH signals between cellular components.^7^ Functional intervention also illustrated how temporal context can change the readout of a single pathway: in experimental SAH, C3aR inhibition reduced aberrant microglial activation, neuroinflammation and white matter injury and cognitive impairment, but intranasal C3a administration during the subacute phase reduced astrocytic reactivity with subsequent improvement in cognitive deficits.^97^ Conclusions that assign complement‐associated signalling as uniformly harmful or protective without specifying the injury phase and modality of intervention should be regarded with caution.

TBI provides additional evidence that microglial responses are modified by injury model, sex, anatomical region and time post‐injury. A murine single‐cell atlas of 334 376 cells from a panel of TBI models (diffuse and contusive), sexes, brain regions and time points identified a disease‐associated microglial lineage expressed at 24 h while transcriptional changes in one microglial population persisted for up to 6 months post‐contusional injury.^98^ More importantly, functional studies also demonstrate that these early microglial population dynamics can dictate recovery. KD025 selective ROCK2 inhibition impaired microglial proliferation and prevented repopulating microglia from replenishing the depleted niche during the early post‐injury period, thus abolishing its purported neuroprotective and cognitive benefits; delaying ROCK2 inhibition to the subacute phase permitted repopulation but did not further enhance those benefits.^99^ Therefore, the TBI data support a functional relationship between persistent transcriptional remodelling and differential timing in microglial repopulation as two correlates of post‐injury heterogeneity.

Global cerebral ischemia following cardiac arrest serves as an additional example of how species, sampling time and functional readout can shape the apparent microglial response. Single‐cell profiling of porcine hippocampus at 6 and 24 h after return of spontaneous circulation in a cardiac arrest model reveals progressive expansion of S100A8‐high microglia together with blood‐brain barrier disruption and neutrophil infiltration, while ligand–receptor analysis implicates neutrophil‐derived resistin in enhanced neutrophil–microglia inflammatory communication.^9^ Additional supporting mouse CA/CPR functional evidence shows that loss of hippocampal dendritic spines was associated with increased numbers of microglia, higher ratios of microglia to dendritic spine contact and enhanced co‐localisation of engulfed spines within CD68‐positive phagocytic vesicles, while treatment with PLX5622 delivered 3 days after CA/CPR partially restored day 6 density of surviving spines.^100^ Collectively, these studies separate an early large‐animal transcriptional and intercellular response from a later mouse functional phenotype of microglia‐associated synaptic loss.

**FIGURE 5:**
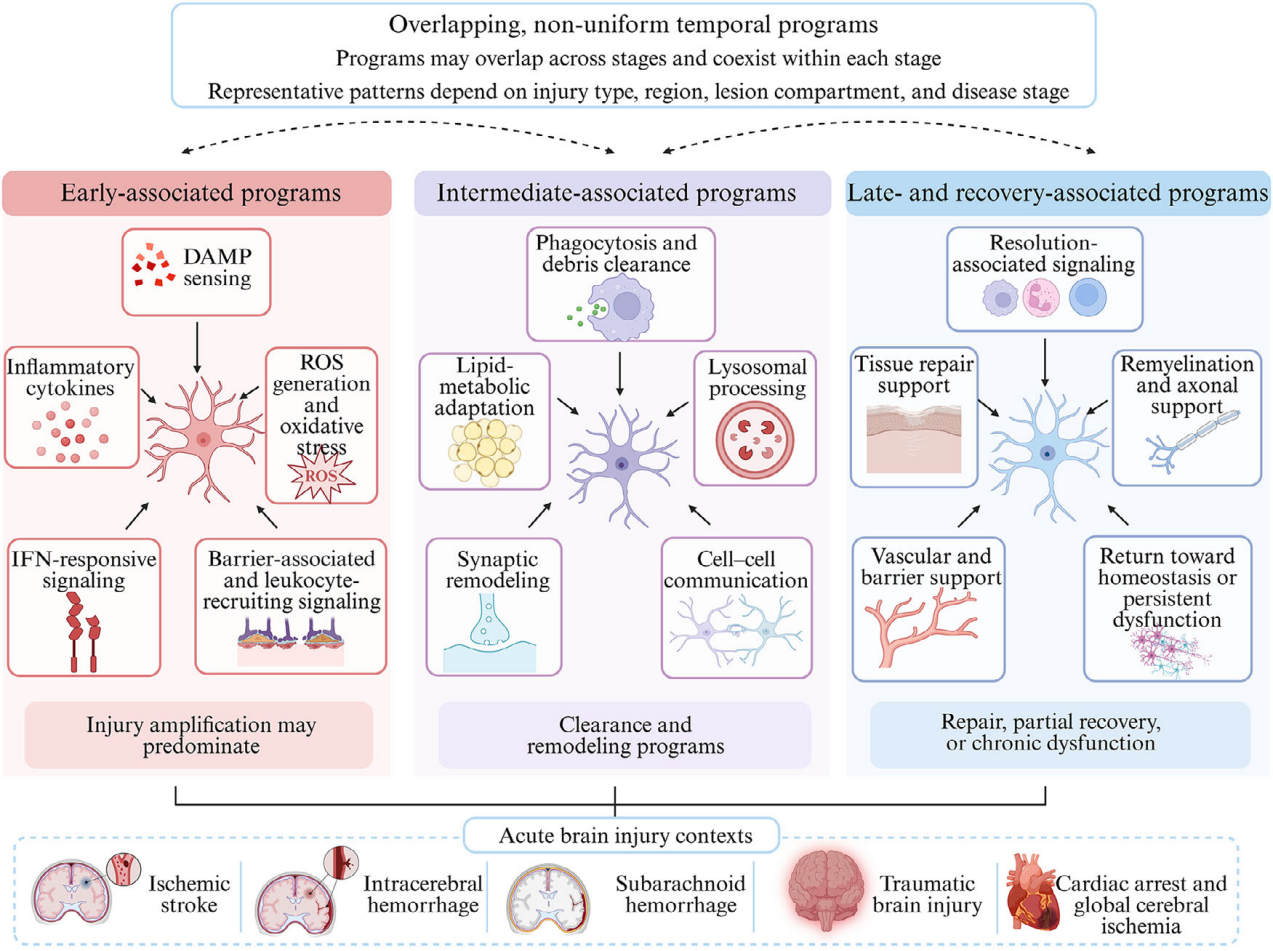
Spatiotemporal remodelling of microglial programs across acute brain injury. The figure shows that microglial responses throughout acute brain injury are overlapping and temporally non‐uniform rather than comprised of discrete activation states over a fixed time course. Responses associated with early events can consist of damage‐associated molecular pattern (DAMP) sensing, inflammatory cytokine production, interferon (IFN)‐responsive signalling, oxidative stress and communication with the compromised blood–brain barrier (BBB) and recruited leukocytes. Increasing participation of phagocytosis and debris clearance, lipid‐metabolic adaptation, lysosomal processing, synaptic remodelling and intercellular communication represents intermediate‐associated programs. Recovery‐associated responses may include resolution signalling, tissue repair, remyelination or axonal support and vascular or barrier support; other persistent dysfunctional programs remain in some situations. The relative contributions and timing of these programs also differ across ischemic stroke, intracerebral haemorrhage (ICH), subarachnoid haemorrhage (SAH), traumatic brain injury (TBI) and global cerebral ischemia after cardiac arrest, as well as depending on the lesion compartment/anatomical region and disease stage.

Thus, acute brain injury does not seem to produce a single conserved pro‐inflammatory microglial state or a universal sequence of advancing states. Instead, the reported programs differ by lesion compartment, species, anatomical region, age or sex, sampling interval and injury model.^101^ Furthermore, the complement of evidence is also quite different in strength; single‐cell and spatial datasets can implicate candidate transcriptional states and intercellular networks, while genetic perturbation establishes pathway‐specific functional requirements.^1^ Pharmacological intervention with temporal control can help to clarify the role of the pathway in pathogenesis over different disease stages. This is especially relevant when delineating therapeutic windows, since a microglia‐directed intervention targeting one set of processes at a given time may have different effects than the same intervention delivered later in disease progression or recovery.^92^, ^102^ Thus, transcriptomic identity alone cannot be used as a sufficient target‐selection criterion. Microglia‐directed interventions will need to couple the molecular target with relevant anatomical compartment, temporal stage and experimentally validated cellular function. Figure 5 now summarises the spatiotemporal coordination of microglial programs in various acute brain injury contexts.

## 4 ROLES OF MICROGLIA IN NEURODEGENERATIVE AND DEMYELINATING DISORDERS

### 4.1 Microglia in AD

AD entails progressive Aβ deposition, tau pathology, synaptic dysfunction and neurodegeneration that together remodel the local microenvironment within which microglia may operate. Microglia are involved in the processing of pathological proteins, inflammatory regulation, metabolic adaptation and communication with surrounding neural and glial cells during disease progression. Single‐nucleus profiling in human tissue also suggests that microglial responses associated with AD consist of different cellular states (which may depend on the experimental region as well as the disease stage), rather than a single disease‐associated phenotype.^103^ Thus, microglial contributions to AD are conceptualised in the context of protein clearance and the interdependent processes of inflammatory and metabolic regulation as well as plaque‐associated intercellular signalling.

Pathological protein handling is an example where the same function of CNS‐resident microglia can be either neuroprotective or neurotoxic depending on receptor status, cargo identity and downstream processing. For example, CRISPR‐mediated deletion of TREM2 in isogenic human iPSC‐derived microglia decreased survival and phagocytosis of non‐self substrates including Apolipoprotein E (APOE), impaired SDF‐1α/CXCR4‐dependent chemotactic activity and attenuated responses to Aβ plaques after xenotransplantation into an AD mouse model.^104^ In turn, the AD‐linked R47H TREM2 variant stimulated binding of phosphatidylserine‐bearing targets and synaptosomes in multiple laboratory microglial systems with R47H‐expressing microglia promoting neuronal killing that was blocked by inhibiting phagocytosis of exposed phosphatidylserine.^105^ A clearer example of cargo‐ and cell‐type‐specific ingestion is complement‐dependent synapse recognition. Deletion of the complement protein C1q reduced astrocytic and microglial synapse engulfment and synapse loss in TauP301S mice, whereas deletion of the Trem2 receptor decreased microglial‐mediated uptake of excitatory synapses but increased removal of inhibitory ones by nearby astrocytes near plaques in TauPS2APP mice.^106^ Bace1 deletion in 5xFAD mice limited to microglia presents a consistent case of accelerated Aβ clearance, combined with improved metabolic reprogramming and autophagolysosomal activity.^107^ These findings suggest that phagocytosis cannot be treated as beneficial or detrimental in isolation; its effects are context‐dependent on the specific identities of the cargo, the engulfing glial cell type, receptor status and downstream lysosomal/metabolic processing.

Microglial clearance capacity in AD inflammatory and metabolic pathways is closely linked. In APP/PS1 mice, Aβ deposition activated NLRP3 while NLRP3 loss increased both glutamine and glutamate metabolism, Slc1a3 expression, mitochondrial activity and α‐ketoglutarate generation with a subsequent increase in Aβ clearance.^108^ The pathway was further corroborated in human cellular systems and pharmacological inhibition reproduced these metabolic and phagocytic effects only after chronic NLRP3 inhibition, suggesting that treatment duration can dictate the functional consequence of inflammasome modulation. Another part of this immunometabolic response is lipid metabolism. Deep analysis of paired mouse AD and human AD single‐nucleus RNA‐sequenced samples revealed that the lipid‐droplet‐associated microglial state enriched for ACSL1 was more likely to be observed in subjects carrying APOE4/4 and that expression of ACSL1 from activated iPSC‐derived microglia could promote triglyceride synthesis, lipid‐droplet accumulation, tau phosphorylation, as well as changes in neurotoxicity responses.^109^ Therefore, changes in metabolism occurring in AD can potentially impact disease through their effects on both pathological protein clearance and the signals released by microglia that are received by neighbouring cells.

The cellular arrangement of the amyloid plaque microenvironment also dictates microglial functions. Using postmortem human tissue, increased phagocytic microglia and astrocyte density within plaques was observed for both cortical and hippocampal regions^110^; however, hippocampal studies were also able to directly correlate the specific morphology of microglia with local patterns of Aβ, pTau and phosphorylated α‐synuclein pathology.^111^ High‐resolution transcriptomic analysis performed using two platforms in a mouse model of AD identified heterogeneous plaque niches where the accumulation of microglia correlated with stronger transcriptional signatures of an astrocytic neurotoxic‐associated response coupled with altered GABAergic and glutamatergic signalling across hippocampal neurons.^12^ These observations suggest that plaque‐associated microglia should be viewed as constituents of a spatially organised multicellular niche rather than solitary responders to Aβ deposition.

Receptor and checkpoint signalling additionally modulate how plaque‐associated microglia convert local pathological signals into inflammatory and phagocytic responses. Astrocytic PD‐L1 inhibition of microglial PD‐1 reduces inflammation and promotes Aβ uptake, while PD‐1 disruption impairs microglial Aβ uptake and CD36 expression and enhances experimental AD by promoting plaque deposition.^10^ TREM2‐associated responses are more complex. While altered TREM2 signalling can disrupt microglial functions in the context of Aβ pathology, sustained TREM2 stability at the cell surface due to reduced receptor shedding accelerates microglial responses and exacerbates neuroinflammation during Aβ pathology.^112^ In conjunction with the cargo‐dependent phagocytic effects observed above, these findings indicate that responses of microglia dependent on their interaction with particular receptors cannot be expressed along a simplistic line from protective to detrimental. These actions are context‐dependent on receptor genotype or availability, cellular ligand source, pathological cargo and also the time point at which the pathway is recruited.

Thus, rather than converging on a single disease‐associated state, AD‐associated microglial responses integrate recognition of pathological proteins and their engulfment via phagocytosis, lysosomal processing of the resulting debris, activation of different types of inflammasomes according to cellular context along with the regulation of multiple aspects of immunometabolism and lipid handling. Deep regional, pathological and genotype‐associated heterogeneity is established by human single‐nucleus, spatial and postmortem studies.^113^, ^114^ The majority of the causal evidence for single pathways is still obtained from mouse models, human iPSC‐derived microglia or engineered cellular systems. Thus, transcriptional assignment to a disease‐associated microglia (DAM)‐like, inflammatory, phagocytic or lipid‐associated state does not determine its functional relevance in the human brain. Boosted phagocytosis could promote the clearance of Aβ when combined with robust lysosomal and metabolic processing, but it can also become harmful when directed towards synapses or stressed neurons. Therefore, even in the case of AD, microglia‐directed strategies will require functional validation of the specific cellular process being targeted and alignment with disease stage, anatomical context and pathological cargo rather than selection arising from a generalised activated or disease‐associated state.^1^

### 4.2 Microglial responses in Parkinson's disease

There is progressive neurodegeneration of nigrostriatal dopaminergic neurons and deposition of pathological α‐synuclein, embedded within a tissue environment that has undergone extensive glial remodelling in the context of Parkinson's disease (PD). Increased nigral microglial abundance, along with expression of immune modulators induced by inflammatory and stress‐related pathways, has been implicated from human single‐nucleus RNA sequencing (snRNA‐seq) of postmortem midbrain in PD.^115^ These measurements give an in vivo basis for the examination of how α‐synuclein handling, inflammasome signalling, autophagic and metabolic regulation, ion‐channel activity and intercellular communication impact microglial function during PD.

The precipitation of α‐synuclein demonstrates a dual relationship between microglial clearance and inflammatory amplification in PD. Fibrillar α‐synuclein provoked microglia to form F‐actin‐dependent intercellular connections that transferred α‐synuclein from overloaded cells to neighbouring microglia, where the cargo was more efficiently degraded; redistribution of the aggregate burden also mitigated inflammatory responses and increased survival of microglia.^116^ In cellular and mouse models, α‐synuclein preformed fibril exposure enhanced PELI1‐dependent LAMP2 degradation, inhibited autophagic flux and promoted the secretion of α‐synuclein‐containing microglial exosomes which seeded aggregation in neurons. The CD11b‐positive exosomes purified from the cerebrospinal fluid (CSF) of PD patients also included α‐synuclein oligomers and encouraged neuronal aggregation.^117^ Moreover, α‐synuclein can amplify inflammasome activity. In models of α‐synuclein preformed fibrils, ASC specks potentiated NLRP3‐dependent microglial activation, reactive microgliosis, α‐synuclein pathology, dopaminergic neurodegeneration and motor impairment; conversely, ASC knockdown inhibited microglial inflammasome activation and reduced neuronal accumulation of α‐synuclein.^118^ These results demonstrate that bystander engagement of microglia with α‐synuclein supports aggregate disposal under conditions where aggregate load is within functional capacity, while lysosomal failure, exosomal release and chronic inflammasome signalling transform cargo processing into mechanisms of pathogenic spread.

A second mechanism by which exposure to α‐synuclein may cause long‐lasting microglial activation is through the regulation of autophagy and lysosomal activity. In a PD model based upon PFF and α‐synuclein, TLR4–STAT1 signalling activation mediated by PFF suppressed transcription of Ulk1 and thus microglial autophagy; this function was restored in the setting of myeloid Stat1 conditional deletion, resulting in further reduction of ULK1‐associated autophagic dysfunction, neuroinflammation, dopaminergic neuronal injury and motor deficits.^119^ Inflammasome turnover is also under the control of CMA. In A53T α‐synuclein models, p38‐dependent suppression of TFEB decreased CMA‐mediated degradation of NLRP3, while either p38 or NLRP3 inhibition inhibited microglial inflammatory responses and dopaminergic neurodegeneration.^120^ This offers an extra pathway of NLRP3 control wherein Parkin promotes K48‐linked polyubiquitination and degradation of NLRP3 in microglia.^121^ However, most of the Parkin evidence came from LPS‐driven experimental systems which are not α‐synuclein‐specific models, thus yielding less confidence that the data directly contributes to sporadic PD‐associated microglial states. Collectively, these studies link impaired autophagic processing to persistent inflammasome activation, while highlighting the variable relevance of each model to disease.

Microglial inflammatory output in PD is further regulated by activity of ion channels and metabolic status. Aggregated α‐synuclein potentiates voltage‐gated potassium channel Kv1.3 expression and activity in primary microglia and experimental PD models, and elevated Kv1.3 expression was also observed in postmortem human PD brain. Genetic Kv1.3 deletion constructs or selective pharmacological inhibition of Kv1.3 function ameliorated α‐synuclein‐induced inflammation and tissue pathology in model systems, showing that Kv1.3 deficiency or inhibition reduced inflammatory responses and neurodegeneration.^122^ The inflammatory environment also exerts influence on metabolic responses. On exposure to PD patient‐derived α‐synuclein fibrils alone and in concert with chronic inflammatory cues, primary microglia exhibited a unique response including perturbation of tricarboxylic acid cycle activity, glutathione and iron metabolism, augmented release of glutamate and resulting toxicity towards midbrain dopaminergic neurons.^123^ The findings show that microglial inflammatory activity in PD is specified through ion‐channel and metabolic mechanisms, rather than by the expression of general inflammatory markers.^124^

Moreover, intercellular communication will decide whether glial responses help clear α‐synuclein or aggravate neuronal injury. Astrocytes in human iPSC‐derived glial cultures transferred internalised α‐synuclein aggregates to microglia by secretory mechanisms and via direct membrane contacts, while aggregate clearance was further enhanced in coculture compared with either cell type alone.^125^ In contrast, α‐synuclein mouse models with partial Cntnap4 deficiency showed that α‐synuclein released from damaged dopaminergic neurons triggered astrocytic C3 production to activate microglial C3aR, which promoted microglial release of C1q and inflammatory cytokines and therefore induced additional death of dopaminergic neurons.^126^ Another microgel‐based proof‐of‐concept for intervention at this communication interface is a delivery system that was shown to reduce oxidative stress and modulate CX3CL1/CX3CR1–NF‐κB–NLRP3‐associated neuron–microglia signalling in experimental PD.^127^ However, this evidence is preclinical and allows for the conclusion that altering intercellular signalling may be feasible using engineered delivery systems but does not demonstrate clinical efficacy.

In a similar manner, PD‐associated microglial responses involve α‐synuclein processing and recognition, activation of the inflammasome, modulation of autophagy and lysosomal functions, ion‐channel functioning, intracellular signalling pathways, metabolic remodelling and cross‐talk with neurons/astrocytes. Nonetheless, the strength of available evidence ranges widely in terms of level of inference and varies according to anatomical region, disease stage and experimental model.^73^ While human single‐nucleus and postmortem studies show disease‐associated microglial alterations with anatomical relevance, most causal associations of NLRP3, STAT1–ULK1, CMA and Kv1.3 are derived from evidence in mouse models of α‐synuclein function, primary or immortalised microglia and engineered coculture systems. Findings from LPS‐ or toxin‐based paradigms should therefore not be assumed to recapitulate α‐synuclein‐driven human PD, and indeed are confounded by the non‐interchangeable nature of these models. Furthermore, increased aggregate clearance is not protective by default when lysosomal capacity is overwhelmed or inflammatory signalling remains active. As such, microglia‐directed strategies in PD must be more clearly aligned with the target (the pathological process itself), cellular interaction, anatomical compartment and disease stage as opposed to merely suppressing a non‐specific inflammatory state.^39^, ^115^

### 4.3 Other neurodegenerative and demyelinating disorders

In addition to working in the context of AD and PD, microglial responses are implicated in a variety of neurodegenerative and demyelinating disorders such as amyotrophic lateral sclerosis (ALS)/frontotemporal dementia (FTD), Huntington's disease (HD), MS and prion disease. These disorders are very heterogeneous in their primary pathology, but microglial functions repeatedly converge on processes of phagocytosis and lysosomal function, regulation of inflammatory responses, synaptic or myelin remodelling and communication with adjacent neural and glial compartments. However, the consequences of these functions depend on pathological substrate, anatomical compartment and disease stage.^128^

Only in ALS and FTD are findings from genomic alterations of disease‐associated genes translated into cellular and functional microglial dysfunction with both cell‐intrinsic effects and additional neurodegeneration‐mediated effects on neighbouring neural cells. Microglia from human iPSCs with VCP mutations demonstrated immune and lysosomal dysregulation, increased inflammatory and phagocytic pathways, and conditioned media from microglial cultures produced Janus kinase (JAK)–STAT signalling activation in human iPSC‐derived motor neurons and astrocytes.^129^ This was extended by human single‐nucleus profiling, which demonstrated genotype‐dependent differences between sporadic and C9orf72‐associated ALS. While sporadic ALS microglia transitioned to disease‐associated states, the state transition of C9orf72 repeat expansion carriers was less pronounced with endolysosomal defects that were reproduced in human microglial xenografts, and patient and C9orf72‐deficient iPSC microglia.^130^ Nonetheless, the associated microglia‐astrocyte ligand–receptor changes were iteratively predicted and localised in silico, and motor‐neuron functional outcomes were not directly assessed. In the SOD1‐G93A ALS model, microglial protein signatures evolved dramatically across disease stages, with late‐stage cells having lower canonical immune function enrichment and greater expression of non‐classical proteins.^131^ Through assessment of these unique phenotypes, it was shown that human iPSC‐derived microglia harbouring the MAPT IVS10+16 FTD mutation demonstrated altered transcriptional states, cytoskeletal abnormalities, a stalled phagocytic pathway, disrupted TREM2/TYROBP signalling and metabolic changes, and together with their secreted factors negatively impacted neuronal synaptic density.^132^ Collectively, these studies show that ALS/FTD‐associated microglial dysfunction can differ depending on the genetic context and could include changes to state transitions and progressive impairment of immune, endolysosomal and phagocytic functions rather than simply hyperactivation.^133^

In HD, microglial responses are tightly connected to each regional synaptic vulnerability. Genetic and postmortem analyses of the HD middle temporal gyrus revealed increased numbers of IBA1‐positive and TMEM119‐positive microglia, somal enlargement, proliferative markers, and heterogeneous reactive morphologies associated with human evidence for cortical microgliosis that did not establish a causal functional role.^134^ Use of isogenic human iPSC‐derived microglia demonstrated a cell‐autonomous role for mutant HTT, increasing basal levels of IL‐6 and IL‐8 secretion or impairing bacterial phagocytosis and transferrin uptake while decreasing late‐endosomes in the absence of further inflammatory stimulation.^135^ Yet, these long CAG‐repeat lengths extended beyond those seen in most adult‐onset HD, and the monoculture design did not lead to neuronal phenotypes. This is more direct evidence for complement‐mediated synaptic engulfment: analysis of human HD tissue revealed specific corticostriatal synapse loss and augmented complement association at affected corticostriatal synapses, whereas in genetic HD models complement was shown to tag these before clearance by microglia; blocking C1q or genetically removing the relevant receptor in microglia can prevent synapse loss and improve early cognitive outcomes.^136^ These results delineate descriptive changes in human microglia, cell‐autonomous dysfunction in vitro and experimentally validated phagocytic pathways in vivo but indicate that clearance‐associated activity can become deleterious when viable synaptic structures are engulfed.

In demyelinating disease, microglia‐mediated phagocytosis can facilitate tissue repair if myelin debris is appropriately recognised and cleared. The expression of TREM2 was particularly upregulated on myelin‐laden phagocytes in active MS lesions, whereas experimental activation of TREM2 promoted myelin uptake and degradation in the cuprizone model, coinciding with an increase in OL precursor density, mature OL formation, remyelination and protection against axonal damage.^41^ In addition, complementary genetic evidence demonstrated that Mertk knockout contributed to impairment in myelin debris clearance and remyelination following cuprizone‐induced demyelination and a different microglial response indicated by scRNA‐seq; this included an increased frequency of type I IFN‐responsive microglia.^137^ Another type of evidence comes in the form of human leukodystrophy tissue. Postmortem analysis of staged white matter in X‐linked adrenoleukodystrophy and metachromatic leukodystrophy revealed loss of TMEM119 and purinergic receptor P2Y12 (P2RY12) expression, yellowing of microglia, low microglial density and injury to phagocytic lysosome or membrane compartments in non‐affected prelesional areas prior to significant OL loss and demyelination.^138^ Since this sequencing was inferred from anatomically staged lesions in seven autopsy cases rather than longitudinal observation, it defines a correlation but not temporal causality. Collectively, these findings suggest that demyelinating disorders may activate receptor‐dependent debris clearance to promote repair or elicit early loss of microglial identity and viability based on disease substrate and stage. However, most causal intervention data are still from experimental demyelination models rather than therapeutic studies in humans.

An example that contradicts this view comes from the study of prion disease in mice where microglial protection does not depend upon reducing the pathological protein burden. In microglia‐deficient Csf1rΔFIRE mice, prion disease was accelerated despite no increase in cerebral prion deposition; however, astrocytes exhibited precocious reactive changes, increased phagocytosis of cytosolic neurons and unfolded‐protein responses.^55^ By contrast, Trem2 deficiency enhanced neuropathology and neuronal loss in the ME7 prion model without modulating the accumulation of disease‐associated prion protein compared with wild‐type mice, and dampened microglial reactivity.^139^ Combined, these data suggest that microglia can promote tissue resilience during chronic proteinopathy via functions at least partially distinct from their role in reducing misfolded protein deposition.

**FIGURE 6:**
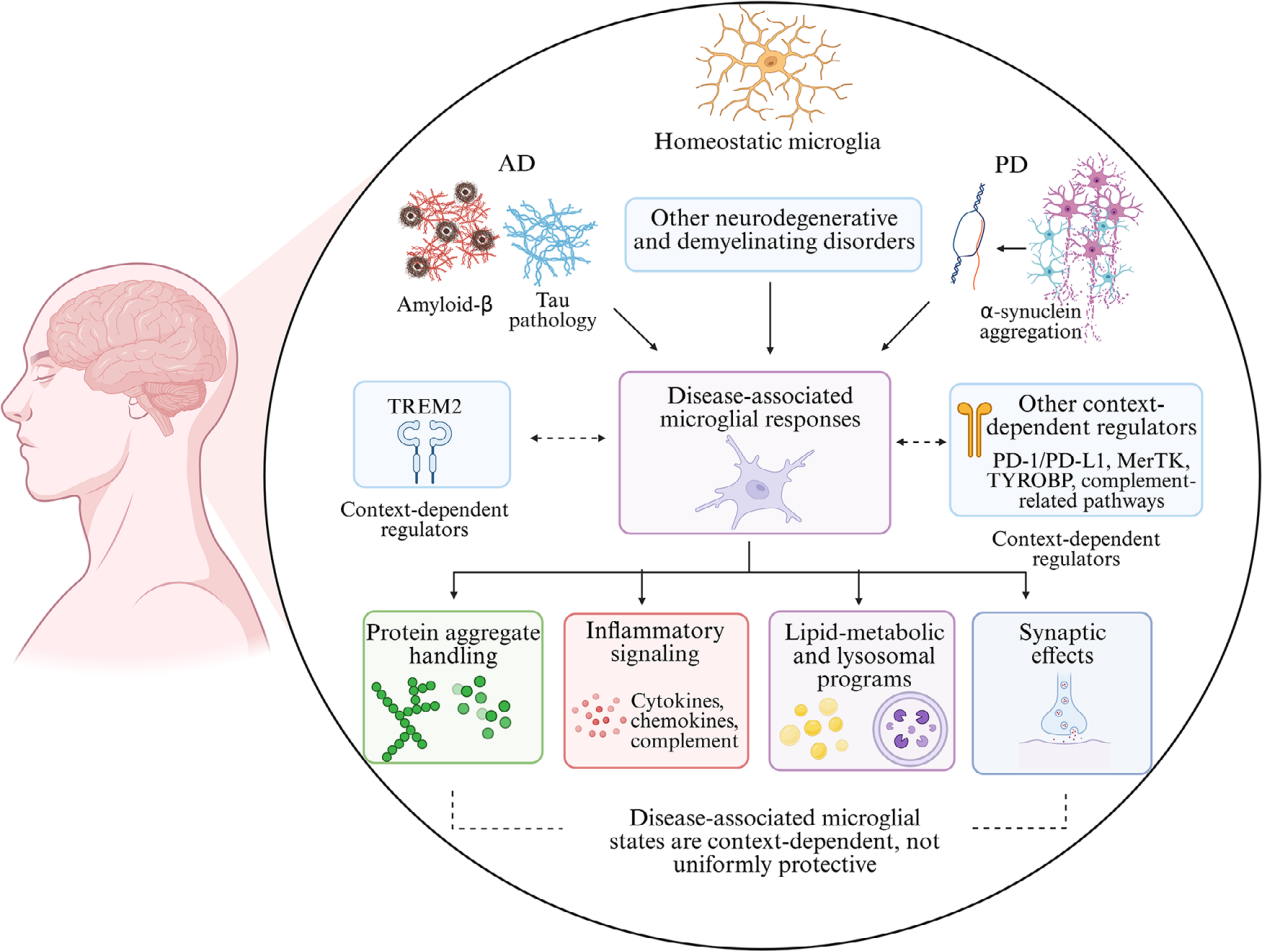
Context‐dependent disease‐associated microglial responses in neurodegenerative and demyelinating disorders. Microglial responses in neurodegeneration and demyelination are modulated by pathological cargo, receptor signalling, disease stage and cell‐intrinsic capacity. Together with pathological processes in other neurodegenerative and demyelinating disorders, amyloid‐β (Aβ) and tau pathology in Alzheimer's disease (AD) and α‐synuclein aggregation in Parkinson's disease lead to heterogeneous microglial responses associated with the respective diseases. Such responses could be significantly affected by TREM2 and other context‐dependent modulators such as the programmed cell death protein 1 (PD‐1)/programmed death‐ligand 1 (PD‐L1) axis, MerTK, TYROBP and complement‐dependent pathways. Large functional classes consist of aggregate and protein signals, inflammatory signalling, lipid‐metabolic and lysosomal functions, and synaptic effects. Lastly, disease‐associated microglia (DAM)‐related transcriptional states should not be interpreted as uniformly protective because their functional impact is influenced by pathological cargo and disease stage in addition to brain region and the ability for downstream lysosomal and metabolic processing.

While some aspects of microglial function converge across neurodegeneration, others diverge when disorders are compared. Disruption of intrinsic immune and lysosomal programs has been implicated in ALS/FTD, complement‐dependent engulfment contributes to synapse loss in HD, efficient clearance of myelin debris supports remyelination, while microglial or TREM2 depletion exacerbates tissue injury without enhancing pathological protein accumulation in prion disease.^55^, ^139^ The available evidence, in terms of lesion properties and biological context, ranges from human postmortem tissue and patient‐derived cellular models to genetically modified rodent systems. As such, transcriptional or morphological assignment of a disease‐associated microglial state does not indicate that the identified function is conserved as protective or pathogenic across diseases.^13^, ^139^ The important outcome relies on pathogenic cargo, anatomical compartment, interacting cellular constituents, stage of disease and the ability to execute phagocytic, lysosomal and metabolic processing. As shown in Table 2, representative disease‐associated microglial programs and functional outcomes as well as the principal limitations of evidence for human relevance are highlighted. Figure 6 summarises the context‐dependent disease‐associated microglial responses across major neurodegenerative and demyelinating disorders.

**TABLE 2:** Microglial programs in neurodegenerative and demyelinating disorders.

Disease	Model/evidence	Microglial functional program	Key pathway(s)	Disease‐relevant outcome	Main limitation	References
**A. Aggregate‐, synapse and lipid‐handling programs**						
AD	TauP301S and TauPS2APP mice with C1q or Trem2 deletion; synaptic multi‐omics and lysosomal analyses	Cargo‐ and cell‐type‐selective synapse engulfment	C1q‐complement tagging; TREM2‐dependent phagocytosis	C1q deletion preserved synapses; Trem2 loss reduced microglial uptake and shifted inhibitory‐synapse engulfment towards astrocytes	Mouse models; whether engulfed synapses were irreversibly dysfunctional was not established	^106^
AD	Human AD cortex stratified by APOE genotype; isogenic human iPSC‐derived microglia	APOE4‐enriched ACSL1‐positive lipid‐droplet state with neurotoxic secretion	APOE4; fibrillar Aβ; ACSL1; triglyceride synthesis	Conditioned medium from lipid‐droplet‐accumulating microglia increased neuronal tau phosphorylation and apoptosis	The secreted neurotoxic factor was unidentified; mechanistic intervention was performed in vitro	^109^
Parkinson's disease	Two α‐synuclein mouse models (AAV substantia nigra overexpression and striatal PFF); primary microglia; peripheral blood mononuclear cells from patients with PD	STAT1‐dependent suppression of microglial autophagy	TLR4–STAT1–ULK1; LC3II/SQSTM1	Microglial Stat1 cKO or ULK1 activation restored autophagy and reduced neuroinflammation, dopaminergic neuronal damage, and motor deficits	Causal efficacy was shown in mouse models; human evidence was limited to peripheral blood rather than brain microglia	^119^
**B. Cell‐autonomous and network programs across proteinopathies**						
C9orf72‐associated ALS	Human motor‐cortex and spinal‐cord snRNA‐seq; human microglial xenografts; patient and C9orf72‐deficient iPSC microglia	Genotype‐specific failure to enter disease‐responsive states with endolysosomal dysfunction	C9orf72 repeat expansion/haploinsufficiency; HLA and disease‐associated programs; lysosomal pathways	C9‐ALS microglia showed weaker disease‐state transitions than sporadic ALS and altered predicted microglia–astrocyte signalling	No direct motor‐neuron functional outcome; ligand–receptor effects were inferred	^130^
VCP‐associated ALS/FTD	Human iPSC‐derived VCP‐mutant microglia; RNA‐seq, proteomics and functional assays with motor neurons and astrocytes	Cell‐autonomous immune and lysosomal dysfunction with non–cell‐autonomous signalling	VCP‐associated immune/lysosomal programs; GPNMB; JAK–STAT	VCP‐mutant microglia showed immune/lysosomal dysfunction; secreted factors activated JAK–STAT in motor neurons and astrocytes	Human iPSC‐based in vitro evidence; no in vivo disease progression or therapeutic benefit was established	^129^
HD	Human Q109/Q22 isogenic iPSC microglia with Q60/Q33 replication	Cell‐autonomous basal cytokine elevation with cargo‐dependent phagocytic and endocytic impairment	Mutant HTT; IL‐6/IL‐8; transferrin endocytosis; late endosomes	Increased inflammatory secretion and reduced bacterial phagocytosis, transferrin uptake and late‐endosome abundance	CAG lengths exceeded most adult‐onset HD; monoculture evidence without a neuronal outcome	^135^
HD	Postmortem HD brain and CSF; two genetic mouse models; C1q blockade and microglial complement‐receptor ablation	Complement‐dependent microglial elimination of corticostriatal synapses	C1q–C3/iC3b–CR3 complement signalling; cortical and striatal mutant HTT	C1q blockade or microglial CR3 loss prevented synapse loss, increased striatal excitatory input and rescued early cognitive deficits	Human findings were associative; causal intervention evidence came from preclinical genetic models	^136^
Prion disease	Csf1rΔFIRE mice lacking microglia after prion infection	Microglial restraint of astrocyte reactivity and neuronal‐material engulfment	CSF1R FIRE enhancer; astrocyte unfolded‐protein response and phagocytosis	Microglial deficiency accelerated clinical disease without increasing cerebral prion accumulation	Lifelong microglial deficiency may alter baseline tissue homeostasis; mouse evidence only	^55^
**C. Demyelination, debris clearance and lysosomal dysfunction**						
MS	Human active MS lesions and TREM2‐deficient macrophages; cuprizone demyelination in mice	TREM2‐dependent uptake and degradation of myelin debris	TREM2–DNAX activation protein 12–SYK; phagocytosis; lipid and cholesterol handling	TREM2 agonism accelerated debris removal, OL‐progenitor accumulation, OL maturation, remyelination and axonal preservation	Cuprizone is an acute toxin model that does not reproduce chronic autoimmune MS; no human intervention	^41^
MS	Mertk‐knockout and wild‐type mice during cuprizone demyelination/remyelination; scRNA‐seq	Mertk‐dependent microglial activation, migration and myelin‐debris clearance	Mertk; IFN‐responsive microglia; IFNγ; phagocytosis	Mertk loss impaired myelin‐debris clearance and remyelination; IFNγ further inhibited clearance and OL differentiation	Mouse cuprizone and knockout evidence; human intervention was not tested	^137^
X‐ALD/MLD	Human postmortem white matter staged across prelesional and demyelinating zones	Early loss of homeostatic identity and microglial viability before major myelin breakdown	TMEM119/P2RY12 loss; DNA fragmentation; lysosomal and membrane injury	Microglial depletion and death preceded OL loss and overt demyelination	Small cross‐sectional autopsy series; temporal sequence was inferred from lesion staging rather than longitudinal observation	^138^

## 5 MICROGLIA AND CNS INFECTIONS

### 5.1 Recognition and clearance of infectious pathogens

Microglia serve as a local immune interface for the detection and response to pathogens in the CNS. They sense pathogen‐related signals, process infectious material by phagocytosis and lysosomal activities, produce antiviral and inflammatory mediators, and cooperate with infiltrating immune cells. These processes are functionally linked but not synonymous: pathogen sensing can initiate inflammatory programs without assuring clearance, while phagocytic uptake does not equate with effective elimination of viable microorganisms.^140^, ^141^ Thus, contributions of microglia to CNS infection should be interpreted within the context of the pathogen, target‐cell population infected and downstream effector function under investigation.

While innate and phagocytic sensing enable both protective factors to limit pathogen proliferation in the CNS, they can work through complementary mechanisms. Here, it is reported that primary murine microglia promptly engaged antiviral and inflammatory transcriptional programs upon neurotropic vesicular stomatitis virus (VSV) exposure, while mitochondrial antiviral signalling protein (Mavs) deficiency governed immune response pathways impairing clearance of the virus in vitro; moreover, Mavs‐deficient mice presented augmented severity and extent of disease from VSV beyond the olfactory bulb.^142^ Bacterial challenge engages a different effector process. Calreticulin and galectin‐3 were similarly released by LPS‐activated primary microglia at the recognition–engulfment interface to opsonise *Escherichia coli* and enhance its phagocytic uptake via LRP1‐ and MerTK‐associated mechanisms.^143^ In primary mouse glial cultures infected with *Staphylococcus aureus*, microglia phagocytosed bacteria and restricted intracellular bacterial survival more effectively than astrocytes at 24 h post‐infection,^144^ allowing for internalisation of *S. aureus* into increased numbers of phagolysosomal compartments. These data suggest that sensing and phagolysosomal processing work through different mechanisms, so that activation or uptake alone does not ensure complete clearance of a pathogen.

Furthermore, pathogen‐derived factors can further instruct whether microglial recognition translates into efficient clearance. However, loss of bacterial neuraminidase A from *Streptococcus pneumoniae* in murine and human microglial systems increased bacterial adhesion but paradoxically promoted intracellular survival, whereas recombinant neuraminidase A contributed positively to phagocytic activation and bacterial clearance.^141^ In the event of fungal infection, a completely different recognition mechanism is activated. Impairment of candidalysin detection through CD11b prolonged cerebral mycosis in experimental *Candida albicans*‐induced cerebral infection, where fungal signals engaged microglial TLR4‐ and CD11b‐associated sensing pathways.^145^ These studies show that pathogen‐specific virulence factors can change both the early recognition of pathogens and the efficiency of subsequent clearance.

This connects resident microglial responses with adaptive antiviral immunity through antigen presentation. In vivo evidence supporting the phenotypic signatures of microglial states and the universal upregulation of MHC class I and IFN signature genes across microglial clusters in CNS myeloid cells isolated from rhesus macaques during acute simian immunodeficiency virus infection was provided by scRNA‐seq, but no direct evidence for pathologically relevant antigen‐specific T‐cell activation was provided.^146^ Direct functional evidence comes from a mouse model of intranasal VSV infection, where microglia acquire viral antigen from neighbouring infected neurons and cross‐present peptide MHC class I complexes to antiviral CD8+ T cells. Whereas microglial depletion reduced antigen‐specific T‐cell calcium signalling and impaired viral control, neuron‐specific MHC class I adhesion did not replicate this.^147^ In a chronic‐infection model, it was shown that the TAP‐dependent MHC class I presentation of *Toxoplasma gondii* antigens from microglia and glutamatergic neurons induced differentiation of parasite‐specific brain‐resident CD8^+^ T cells.^148^ Collectively, these studies differentiate transcriptional signatures of antigen‐presentation ability from experimentally verified antigen‐specific interactions and suggest that local presentation of exogenous antigens links pathogen‐derived material to adaptive immune responses in the context of both acute and chronic CNS infections.

Thus, recognition of pathogens, phagocytosis and antigen presentation can each be considered as separate but interacting functions of the microglial response to CNS infection. The relative importance of various factors differs for viral, bacterial, fungal and parasitic pathogens and depends on whether microglia encounter free microbes in tissue, infected neural cells or pathogen‐derived material (e.g., debris). The strength of the evidence available also varies widely: studies targeting a genetic perturbation or probing antigen‐specific interaction analyse functional requirements in defined experimental systems, whereas most single‐cell studies in non‐human primate and murine infection models characterise infection‐associated transcriptional states.^140^, ^149^ In addition, successful pathogen uptake does not equate to pathogen elimination and inflammation beneficial for early control of the pathogen may prove deleterious if prolonged. Collectively, these data suggest that microglial responses in CNS infection must be interpreted specifically according to pathogen class and cellular target, disease stage and experimentally demonstrated effector function rather than under the umbrella of a generally protective antimicrobial state.

### 5.2 Roles of microglia in viral encephalitis

In viral encephalitis, microglia contribute to antiviral defence but also respond to tissue damage and inflammatory mediators within the infected CNS. These functions comprise alterations in transcriptional programs, direct or indirect sensing of viral infections, cytokine production, interactions with neurons, astrocytes and peripheral immune cells, phagocytic clearance and control of metabolic homeostasis. Consequently, their functional consequences are not only dictated by the level of microglial activation but also by tropism, the infected cellular compartment, timing of response and effector processes involved.^150^, ^151^

Viral infection induces heterogeneous microglial responses with an interpretation of activation depending on the experimental strategies used to describe them. A VSV model combined with microglia‐specific translatomic analysis revealed that programs associated with cytokine production and T‐cell activation were enriched in the context of infection, whereas this study also showed that besides isolation, cell sorting alone induced cell‐driven transcriptional artifacts.^152^ Human microglia are also directly susceptible to, and respond differently to, neurotropic viruses. Long‐term productive infection by three strains of tick‐borne encephalitis virus and the strain‐dependent associations between viral replication, cytokine and chemokine production, and cellular ultrastructure were observed in primary human microglia and in microglial cell lines.^153^ These results demonstrate that viral encephalitis‐associated microglial states are both pathogen‐specific and dependent on the analytical paradigms and criteria used to measure and interpret them, and transcriptional activation should not be interpreted as a specific antiviral or pathogenic effector without other supporting evidence.

Cell‐intrinsic pathways and signals from neighbouring neural cells regulate antiviral signalling in microglia. Reduced expression of miR‐590‐3p increased USP42, stabilising TRIM21 and thus enhancing the OAS1‐associated antiviral signalling pathway via deubiquitinases, linking a microRNA‐deubiquitinase pathway to the microglial response to infection in Japanese encephalitis virus (JEV)‐infected human microglial cells.^154^ In marked contrast, VSV encephalitis displays a critical non–cell‐autonomous mechanism wherein experiments on conditional disruption revealed that microglial accumulation and activation were regulated by type I IFN receptor signalling in neurons and astrocytes, not microglia themselves, which ultimately determined susceptibility to infection.^155^ These studies separate intracellular antiviral control from intercellular regulation of the microglial response, leading to the conclusion that microglial activation during viral encephalitis can be influenced heavily by signals from infected or otherwise activated neural cells.

Herpes simplex encephalitis provides another example of virus‐induced inflammatory responses that are both protective and potentially deleterious in the same disease. In a mouse model of herpes simplex virus 1 (HSV‐1) neuroinvasion, depletion of microglia led to a higher viral burden in the brain and increased severity of disease, while cyclic GMP–AMP synthase (cGAS) deficiency exacerbated disease,^156^ suggesting early cGAS‐associated microglial antiviral responses that involved type I IFN production. On the other hand, a different result can be produced by sustained or a certain type of metabolically dysregulated activation. In another HSV‐1 model that underwent gut microbiota depletion, microbial metabolites like nicotinamide N‐oxide restored NAD^+^‐dependent mitophagy and reduced microglia‐associated inflammatory responses to alleviate HSE.^157^ Together, these findings indicate that the timing and metabolic state of responding microglia may modulate their contribution from early augmentation of antiviral restriction to late‐phase inflammatory tissue injury, but supporting mechanistic intervention data are still required.

Assessing pathway‐selective perturbations in mouse models of viral encephalitis, evidence indicates that even when host defence is not impaired at the population level by microglia depletion, individual microglial programs operate with pathological consequences for the course of neurological injury. PLX5622‐mediated microglial depletion also prolonged viral persistence, accelerated onset of seizures and worsened hippocampal and spinal cord pathology in Theiler's murine encephalomyelitis virus (TMEV)‐infected mice.^158^ However, specifically in TH2‐polarised microglia, Ido2 deficiency reduced pathological effects caused by a defined microglial pathway in promoting disease progression.^159^ In flaviviral encephalitis, common population‐level protection was seen as PLX5622‐mediated depletion increased viral titres and mortality in the brains of mice infected with West Nile virus or JEV.^160^ In an immunocompromised mouse model of Zika virus infection, microglia displayed enhanced phagocytic and extracellular digestive activity while their depletion resulted in significantly greater viral load within the brain and partial compensatory phagocytic activity by astrocytes.^161^ Collectively, these data help explain why global deletion of microglia should not be viewed as equivalent to selective blockade of any one inflammatory or metabolic pathway.

Consequently, the nature of the particular viral‐related insult does not evoke a stereotypical, conserved microglial response. Microglia can both inhibit viral replication and orchestrate antiviral immunity, although certain inflammatory or metabolic programs may contribute to seizures or tissue damage.^140^, ^158^, ^159^ Such effects are dependent upon viral type, disease stage, infected cellular compartment and specific microglial function involved and urgently warrant distinguishing protective antiviral defence in neurons from inflammation generally associated with neurological injury.

### 5.3 Chronic infection and persistent inflammation

Unlike the acute syndrome of encephalitis, chronic CNS infection can be sustained by pathogen persistence, latent reservoirs and incomplete resolution of immune activation, allowing for continued microglial reactivity long after the actual infecting agent has been eliminated from the parenchyma. In this microenvironment, microglia may participate in the generation of persistent pathogen reservoirs and continuous inflammatory signalling.^162^, ^163^ However, the long‐term fate of these responses, whether they are sustained in a chronically altered state or return towards homeostasis, is determined by the biology of specific pathogens, treatment status, interactions with infiltrating immune cells and persistence of pathogen‐derived stimuli.

The implication from human immunodeficiency virus (HIV) that microglia are a long‐lived reservoir can be further extrapolated to suggest that they contribute directly to pathogen persistence in the CNS. Isolated brain myeloid cells were largely TMEM119‐positive microglia from non‐human primates, while rapid‐autopsy samples from ART (antiretroviral therapy)‐treated people with HIV contained both total and integrated viral DNA; furthermore, inducing replication‐competent virus recovery was achieved in parietal‐cortex microglia from one such treated subject.^162^ Additional supporting experimental evidence stems from a humanised mouse model in which xenografted microglia were infected with HIV‐1 and genetically traced in vivo to better characterise the mechanisms underlying CNS HIV infection and latency.^164^ Collectively, these studies separate a direct demonstration of an enduring human brain reservoir from an experimental apparatus poised to interrogate cellular and genetic mechanisms governing the reservoir.

Even in the setting of systemic infection control, inflammatory activity can persist with viral reservoirs. In virally suppressed people with HIV, IFN‐inducible Mx1 and TNFα were increased in brain myeloid cells by quantitative RT‐PCR of postmortem frontal cortex relative to HIV‐seronegative controls, and the number of Mx1‐positive myeloid cells was correlated with total, intact and defective proviral DNA.^165^ In a study of human iPSC‐derived cerebral and choroid‐plexus organoids, HIV‐infected microglia produced CCL2, CXCL10, type I IFN‐stimulated genes and S100‐family inflammatory programs; low‐level chemokine production persisted after ART with correlative bystander inflammatory changes in the cells.^166^ These results link viral persistence with ppRNA‐mediated elevation in neuroimmune signalling that is associated with, but separable from, microglia‐driven effects based on experimentally defined outcomes in human postmortem tissue.

Persistent infection is not synonymous with irreversible microglial inflammatory programs. PACAP administration attenuated microglial activation, monocyte recruitment, inflammatory mediator expression and neuronal damage in a chronic *Toxoplasma gondii* mouse model, providing preclinical evidence that PACAP could modulate infection‐associated neuroinflammation in the brain without establishing clearance of the pathogen reservoir.^167^ In another *Trypanosoma brucei* infection model employing both fate mapping and single‐cell sequencing, whereas recruited monocyte‐derived macrophages underwent rapid clearance following resolution of disease, resident microglia gradually reverted towards a homeostatic state.^168^ These findings suggest that the programming of chronic inflammation and pathogen persistence is a related but separate process, and that reversible or irreversible states of microglia are contingent on resolution of infectious and inflammatory stimuli.

**FIGURE 7:**
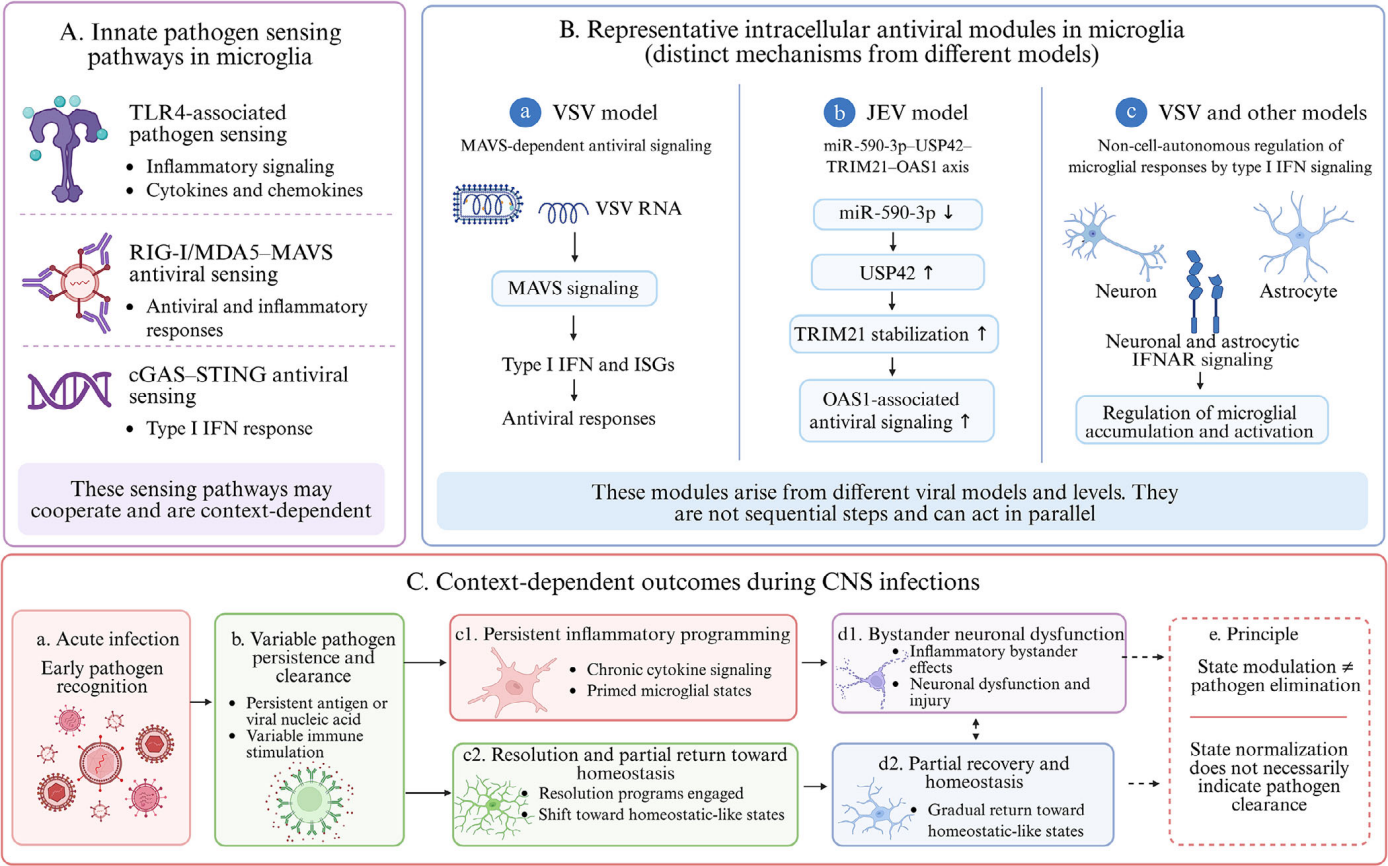
Context‐dependent microglial responses during central nervous system (CNS) infection. (A) Microglia are activated via several innate pathogen‐sensing pathways during CNS infection. Signalling through TLR4 is associated with inflammatory responses and cytokines; signalling through mitochondrial antiviral signalling protein (MAVS) supports antiviral responses; and sensing by cyclic GMP–AMP synthase (cGAS) promotes type I interferon (IFN) signalling. (B) Distinct intracellular and non–cell‐autonomous antiviral mechanisms are indicated by viral models here and should not be interpreted as sequential steps. MAVS‐dependent signalling promotes type I IFN and antiviral responses during vesicular stomatitis virus (VSV) infection. Reduced miR‐590‐3p increases USP42 in Japanese encephalitis virus (JEV)‐infected microglia to stabilise TRIM21 and supports OAS1‐associated antiviral signalling. In the context of VSV and other types of experimental settings, type I IFN signalling in neurons and astrocytes can alter microglial accumulation and activation. (C) CNS infection may take distinct context‐dependent trajectories. These trajectories evolve along a cardinal axis from acute pathogen response towards either pathogen persistence and sustained inflammatory programming or towards partial resolution back to homeostasis. Chronic inflammatory signalling can lead to bystander neuronal dysfunction, while the ability of anti‐inflammatory modulation of the microglial inflammatory state may not clear ongoing persistent pathogens or reservoirs. These trajectories are determined by pathogen biology, host factors (e.g., genetics, age), microenvironment and disease stage.

Thus, sustained pathogen persistence can be associated with long‐term inflammatory programming of the CNS following chronic infection, even though these processes are separable. Microglia can retain pathogen debris, transduce inflammatory signals to neighbouring tissue or revert towards homeostasis after resolution of the disease. This distinction between reservoir‐directed and inflammation‐modulatory effects is essential for therapeutic interpretation, particularly in evaluating how an intervention affects not only pathogen burden but also microglial function.^162^, ^169^ Figure 7 summarises context‐dependent microglial responses across pathogen sensing, viral encephalitis and persistent CNS infection.

## 6 MICROGLIA IN PSYCHIATRIC AND NEURODEVELOPMENTAL DISORDERS

### 6.1 Microglial mechanisms in depression and anxiety disorders

Microglial dysregulation in depression and anxiety is more accurately conceived of as context‐dependent alterations in inflammatory signalling, metabolism, phagocytosis and intercellular communication (which often overlap substantially) than as a uniformly activated phenotype. While these functional programs can be remodelled by stress, peripheral metabolic cues and challenges to immune homeostasis, the adaptations and outcomes of such remodelling are region‐, stage‐ and disease‐dependent. This means that mechanistic findings of experimental stress models do not necessarily translate to human observations, and changes in microglial markers alone should not be seen as evidence of a specific pathogenic function.^170^, ^171^

There is no evidence of a global hyperinflammatory activation of microglia during major depressive disorder (MDD). RNA sequencing (RNA‐seq) of acutely isolated microglia from postmortem occipital grey matter and corpus callosum of 13 donors with MDD and 10 matched controls revealed 92 differentially expressed genes in grey matter microglia but none in white matter microglia. The majority of grey matter changes were associated with decreased expression of immune and phagocytosis‐related genes, such as complement‐associated genes, but an increase in synaptic CD47 protein.^172^ Conversely, a different study found that the prefrontal cortex from individuals with MDD displayed upregulated GLS1 and neuroinflammation‐related pathways, and postmortem inhibition of microglia‐specific Gls1 in mice subsequently reduced LPS‐induced inflammatory responses as well as anxiety‐ and depression‐like behaviours along with astrocyte reactivity.^173^ Together, these findings illustrate the regional and analytical specificity of microglial dysregulation in depression while distinguishing human descriptive molecular alterations from cell‐type‐specific causal proof in experimental models.

Both peripheral metabolic and local trophic signals provide complementary circuits for how stress may influence microglial function.^174^ Rifaximin also changed gut microbiome composition and short‐chain fatty acid levels in CUMS adolescent rats, accompanying the changes in hippocampal microglial inflammatory profiles and neurogenesis.^175^ Particularly, a unique systemic metabolic pathway was identified in CUS‐challenged mice which involved running‐induced increases in circulating adiponectin and AdipoR1 in the hippocampus associated with modification of inflammatory responses coupled to AMP‐activated protein kinase (AMPK)‐, NF‐κB‐ and STAT3‐signalling pathways. These effects were attenuated by hippocampal AdipoR1 knockdown and reproduced several microglial and behavioural abnormalities associated with stress.^176^ A hippocampal complementary mechanism with opposed effects was demonstrated in chronic mild‐stress mice where microglial IL4R knockdown decreased Arg1‐positive microglia, BDNF‐dependent neurogenesis and stress resilience, while hippocampal IL4 delivery increased neurogenesis and decreased stress‐induced depression‐like behaviour.^177^ Taken as a whole, these studies demonstrate the potential for peripheral physiology and local cytokine–neurotrophin signalling to remodel stress responses, but corresponding evidence in humans remains sparse and does not show that similar interventions will alter human depression.

Here, the effects of innate immune signalling and phagocytic activity diverge according to the material being engulfed and when stress exposure occurred. In chronic restraint stress mice, stimulator of IFN genes activation with 2′3′‐cGAMP increased microglial phagocytosis and decreased TNF‐α, IL‐6 and IL‐1β production and depression‐like behaviours.^178^ In turn, prior inflammatory challenge in early life followed by adolescent stress upregulated CX3CR1‐dependent microglial engulfment of glutamatergic dendritic spines within the anterior cingulate cortex; this depressive behaviour was also prevented by microglia depletion or inhibition.^179^ Another mechanism involves signalling from neurons to microglia. In MDD, serum exosomal miR‐9‐5p was elevated, while cell and mouse experiments indicated that neuron‐derived miR‐9‐5p inhibited microglial SOCS2, stimulated the JAK/STAT3 signalling pathway and increased production of inflammatory cytokines.^180^ Importantly, these data illustrate that increased microglial phagocytic or inflammatory activity cannot be regarded as uniformly beneficial or harmful, with outcomes resting upon upstream signalling, phagocytic cargo, developmental stage and neuronal context.

Indeed, human challenge and patient‐derived‐cell studies suggest that the exaggerated neuroimmune activation seen in some other disorders need not apply to affective and anxiety‐related disorders. In 15 participants with PTSD and 15 matched controls, LPS induced a significantly smaller increase in [11C]PBR28 TSPO availability within the prefrontal‐limbic circuit in the PTSD group, and greater suppression of this neuroimmune response was associated with more severe anhedonia.^181^ This finding supports suppression of inducible neuroimmune responsiveness rather than a microglia‐specific mechanism since TSPO is also expressed by astrocytes and endothelial cells, and again the sample is small. Microglia‐like cells from patients with panic disorder (*n* = 17), used as a proxy for intrinsic immune function, displayed lower levels of phagocytosis, higher TREM2 and TDAG8 expression and abnormally low ACAT2‐ and DHCR7‐related cholesterol‐biosynthesis programs compared to controls (*N* = 16).^182^ Induced cells are not resident brain microglia, challenging their relation to in vivo panic circuitry. Transcutaneous auricular vagus nerve stimulation attenuates hypothalamic microglial expression of inflammatory markers in CUMS‐exposed rats via α7nAChR‐, JAK2‐, STAT3‐ and NF‐κB‐associated signalling; these effects were significantly blunted in α7nAChR‐deficient rats.^183^ Together, these studies separate human dynamic or patient‐derived associations from pathway‐specific preclinical perturbation, arguing against a universal amplified state of microglial inflammation.

Microglial alterations across stress‐related and depressive‐like disorders do not converge onto a uniform pro‐inflammatory state. Human studies and patient‐derived models reveal cell type‐ and clinical‐context‐dependent molecular, metabolic and phagocytic impairments, while in vivo work shows that metabolic/inflammatory signalling, synaptic engulfment and intercellular communication can regulate depression‐ and stress‐related behaviours through distinct mechanisms.^170^, ^171^, ^184^ This necessitates the separation of human association from model‐specific causality and identification of the cellular source, microglial function, brain region and upstream stressor involved in therapeutic interpretation.

### 6.2 Microglial mechanisms in schizophrenia

Microglial dysfunction in schizophrenia is not confined to diffuse neuroinflammation. Human postmortem studies reveal considerable diversity across inflammatory cohorts while human microglia‐like cellular systems and genetic perturbation experiments point to complement‐dependent synaptic crosstalk and metabolic control.^185^, ^186^ Thus, interpretation depends on separating increases in microglial density or marker expression from established cell function and classifying resident microglial responses as distinct from those driven by macrophages.

The microglial and myeloid evidence is schizophrenia‐subgroup dependent and heterogeneous across analytical approaches in human postmortem studies. In dorsolateral prefrontal cortex from 72 individuals with schizophrenia and 69 controls stratified by inflammatory status, most microglial transcripts were unchanged or decreased in the high‐inflammation schizophrenia subgroup; however, macrophage‐associated CD163 and CD64 and the recruitment chemokine CCL2 were increased.^187^ Moreover, when examining nine independent transcriptomic datasets, decreased expression of a previously defined microglial signature gene was detected in several brain regions including cerebellum, associative striatum, hippocampus and parietal cortex, although the bulk‐tissue data cannot define an exact functional state for individual microglia.^188^ In a further postmortem prefrontal cortex study on 62 matched schizophrenia‐control pairs, increased expression of C4 and C1q was accompanied by the microglial phagocytosis‐associated genes AXL, MERTK and CD68.^189^ These results reject the concept of a unitary inflammatory microglial phenotype and reveal that factors including cellular source, inflammatory subgroup, brain area and molecular readout can substantially shape the perceived immune profile of schizophrenia.

Signalling through complement is the mechanistic bridge between synapse engulfment and schizophrenia risk, though confidence in each experimental system informing one another is limited. Humans genetically encode four isoforms (two protein‐coding genes) of C4A and C4B, which influence their binding to synapses: in mice expressing human C4A, C4A exerted more efficient synapse binding than did its closest relative, C4B and overexpression of human C4A increased random microglia engulfment of synaptic material, reduced the density of cortical synapses and modified behaviour.^190^ Additional support from human iPSC‐derived neuron and microglia‐like cell binary cultures demonstrated that schizophrenia‐derived microglia‐like cells had upregulated NF‐κB‐ and inflammasome‐related activity as well as caspase‐1 activation paired with increased synapse loss; anti‐inflammatory pretreatment diminished both microglial activity and abnormal synapse loss.^191^ A human cellular model (due to anonymisation of patient‐derived monocyte‐induced microglia and iPSC‐derived neural material) showed more synapse uptake in schizophrenia‐derived than control cells from the human brain, only partially determined by C4 risk variation, with minocycline significantly reducing uptake in vitro.^192^ It is emphasised that induced microglia are not resident brain microglia and the associated electronic health‐record association is inconclusive for prevention here. Collectively, these studies tie complement and inflammatory signalling to microglia‐mediated synaptic dysfunction in the context of schizophrenia, while existing genetic mouse models alongside patient‐derived cellular systems fail to support generalised overstimulation of phagocytic activity within the human schizophrenia brain.

Additional factors, including metabolic and intercellular signals, dictate whether microglial synapse processing is excessive or dysfunctional. Specific to the synaptic disease, in patient‐derived neuronal and microglial cultures and forebrain organoids, kynurenic acid increased microglial internalisation of synaptic structures while inhibition of endogenous kynurenic acid production decreased synaptic uptake; integrated genetic and postmortem analyses provided further support linking kynurenine aminotransferase‐associated networks with synaptic activity and schizophrenia genetic risk.^193^ In human iPSC‐derived glial cultures, however, TNF‐α‐stimulated astrocytes isolated from schizophrenia subjects induced a microglia‐like cell dystrophic or senescent program, reduced synaptoneurosome phagocytosis and altered migration along the CX3CL1–CX3CR1 axis.^194^ Together, these results suggest opposite combinations of excessive synaptic engulfment and impaired phagocytic competence in schizophrenia‐associated microglial dysfunction dictated by upstream metabolic/glial signals.

Thus, microglial changes associated with schizophrenia are not simply in a single activated state but instead span programs of inflammation, phagocytosis and dystrophy. Functional studies with genetic and patient‐derived cellular models suggest that there is complement‐dependent microglia‐synapse signalling as well as impaired phagocytic function and glial‐interaction mechanisms.^194^, ^195^ Thus, translation will ultimately depend on elucidating if altered microglial activity is causal or merely a consequence of primary disease processes and whether synaptic perturbations reflect aberrant microglia‐synapse interplay or impaired phagocytic clearance.

### 6.3 Microglial mechanisms in autism spectrum and related neurodevelopmental disorders

The contributions of microglia to autism spectrum disorder (ASD) and other neurodevelopmental disorders must be interpreted in a context‐dependent manner, depending upon developmental timing, sex, brain region, immune status and the functional cellular phenotypes affected. Microglia play a role in synapse recognition and elimination in neurodevelopmental models such as ASD, but their dysfunction may be that of inadequate synaptic pruning and phagocytosis rather than an increase in inflammation across the board.^196^, ^197^ Different microglial programs of inflammation, phagocytosis and synaptic function may be implicated in related disorders. Such human molecular observations must thus be viewed separately from causal data derived from genetic perturbation and experimental models of maternal immune modification.

Human brain profiling supports molecular modifications of microglia in ASD, but functional implications await further work. Deep snRNA‐seq of postmortem ASD cortex, verified via single‐nucleus chromatin accessibility and spatial profiling, detected cell‐type‐specific transcriptional changes in addition to transitions from homeostatic to reactive states in microglia and astrocytes.^198^ These data contextualise microglial changes within a multicellular cortical response and provide human evidence for altered glial states, but the transcriptomic state assignment is insufficient to approximate whether microglia excessively prune synapses, fail to clear them or alternatively are responding secondarily to neuronal pathology.

Dynamically shifting risk mechanisms are evident with genetic ASD‐associated perturbations that align with excessive synaptic elimination via increased microglial phagocytic activity. In Scn2a‐deficient mice, microglia were found to be excessively pruning postsynaptic structures in a complement C3‐associated manner during specific developmental windows, while microglial depletion partially corrected synaptic transmission and spine density; increased postsynaptic elimination was also modelled by recapitulating iPSC‐derived human microglia incorporated into cerebral organoids carrying an ASD‐linked SCN2A mutation.^199^ An orthogonal CRISPR interference screen performed in human iPSC‐derived microglia‐neuron coculture identified ADNP as an ASD risk gene controlling microglial endocytosis and synaptic pruning.^200^ C1q‐dependent microglial synaptic pruning in the medial prefrontal cortex was increased in Msn‐knockout mice, associated with synaptic deficits and impaired social novelty, but prevented by treatment with IFN alpha and beta receptor (IFNAR)1 blockade that reversed both the microglial abnormalities and behavioural impairment.^201^ Non‐phagocytic microglial programs may also be implicated in related neurodevelopmental risk. Microglia‐specific Mecp2 deficiency activates RIPK1‐dependent oxidative stress, cytokine production and SLC7A11/SLC38A1/GLS‐associated glutamate release in a murine model of Rett syndrome (RTT), and genetic or pharmacological RIPK1 inhibition in Mecp2‐null mice ameliorates excitatory neurotransmission and motor dysfunction and prolongs survival.^202^ Since disease‐modifying experiments were done mainly in male mouse models, the findings may not be relevant in human RTT, which is primarily a female disorder. In sum, these studies suggest that neurodevelopmental genetic risk influences synaptic remodelling and inflammatory signalling or neurotransmitter‐related microglial functions via neuron‐initiated and/or microglia‐intrinsic pathways.

On the other hand, synapse elimination is also required for developing circuits. Tmem59 loss of function specifically in microglia prevents synaptic engulfment through destabilisation of the C1q receptor CD93, increases excitatory synaptic density and transmission and causes ASD‐like behaviours in mice.^203^ Lastly, another developmental recognition mechanism is TREM2: Trem2 deficiency leads to less microglia‐mediated synapse pruning and more excitatory neurotransmission, long‐range functional connectivity disruption, repetitive behaviour and altered sociability in knockout mice; the protein levels of TREM2 correlated negatively with severity of symptoms in autism patients.^204^ A similar outcome is reached when checkpoint regulation is taken into consideration. In 16p11.2 deletion mice, the generally increased neuronal CD47 was connected with microglial phagocytosis as well as increased excitatory synapses.^40^ These results indicate that aberrant microglial function associated with ASD should not necessarily be considered as excessive pruning, because reduced synaptic elimination (leading to an accumulation of excess and damaged spine material) can produce a dissociable pathophysiological pathway to circuit dysfunction.

Environmental and maternal immune signals provide additional evidence that the direction of microglial synaptic remodelling is context‐dependent in development. Altered gut microbiota promoted microbe‐derived indole‐3‐propionic acid (IPA) depletion, disrupted IPA/aryl hydrocarbon receptor/NF‐κB signalling and hippocampal microglial overactivation in a prenatal caffeine exposure‐induced intrauterine growth restriction model with evidence for excess postnatal synaptic loss; reduced serum IPA was associated with children exhibiting intrauterine growth restriction features whereas the corresponding link between causative microglial activation and behaviour was revealed based on implications from their rat model.^205^ Maternal LPS exposure is a unique inflammatory stimulus. Prenatal exposure to LPS was followed by TLR4/NF‐κB‐associated inflammatory signalling, along with increased microglial and synaptic measures and ASD‐like behaviours in offspring of wild‐type but not Tlr4‐deficient mice.^206^ This experiment supports a role of TLR4‐dependent maternal–foetal inflammatory signalling but does not confirm a microglia‐specific TLR4 mechanism because Tlr4 deficiency was not restricted to microglia. In a contrasting finding, maternal PRRSV infection, but not postnatal microglial MHC II expression or LPS‐evoked cytokine production,^207^ reduced sociability in neonatal piglets within a large‐animal maternal‐infection model. Its negative result thus suggests that maternal‐infection‐associated social behaviour does not depend on continued postnatal microglial immune activation and cautions against assuming conservation across species, pathogens and developmental sampling windows.

Microglial dysfunction in ASD and related neurodevelopmental disorders cannot be summarised along an inflammatory or phagocytic axis. Thus, ASD‐associated genomic and environmental perturbations can either drive excessive pruning or insufficient pruning through complement, phagocytic receptors, phagocytic checkpoints and inflammatory signalling.^197^, ^199^, ^208^ Rett syndrome is characterised by loss‐of‐function mutations in the X‐linked MECP2 gene which, through its post‐translational effects on MeCP2 protein expression, engages many pathways including RIPK1‐dependent oxidative and glutamate‐release programs. Human transcriptomic studies largely associate altered cellular states without delineating causal mechanistic functions; nor do cross‐species maternal‐infection models reveal an evolutionarily conserved correspondence between social behaviour and persistent lifelong microglial activation. For interpretation, the microglial mechanism must be aligned with its developmental window, brain region, genetic or environmental context and the cellular function measured.^196^, ^202^, ^209^ A summary of representative microglial programs, evidence contexts, functional outcomes and key limitations across psychiatric and neurodevelopmental disorders is shown in Table 3.

**TABLE 3:** Microglial programs in psychiatric and neurodevelopmental disorders.

Disorder	Model/evidence	Microglial functional program	Key pathway(s)	Neural/behavioural outcome	Main limitation	References
**A. Mood‐, stress‐ and fear‐related programs**						
MDD	Postmortem prefrontal cortex from individuals with depression; microglia‐specific Gls1 cKO mice in an LPS‐induced depression model	GLS1‐dependent microglial inflammatory signalling and microglia‐to‐astrocyte communication	GLS1; extracellular‐vesicle miR‐666‐3p/miR‐7115‐3p–Serpina3n signalling	Microglial Gls1 deficiency reduced pro‐inflammatory cytokines and astrocyte reactivity and attenuated LPS‐induced anxiety/depression‐like behaviour	Human evidence was postmortem and associative; causal intervention used an acute LPS mouse model	^173^
Stress‐related depression	Chronic mild‐stress mice with microglial IL4Rα knockdown or hippocampal AAV‐IL4	IL4‐driven microglial support of neurogenesis and stress resilience	IL4‐IL4Rα/STAT6; microglial BDNF‐TrkB	IL4‐driven microglia restored hippocampal neurogenesis and stress resilience	Mouse evidence; the Arg1‐positive state and AAV‐mediated intervention do not establish efficacy in human depression	^177^
Post‐traumatic stress disorder	TSPO PET before and 3 h after intravenous LPS in 15 participants with Post‐traumatic Stress Disorder (PTSD) and 15 controls	Suppressed inducible neuroimmune response	LPS‐evoked TSPO dynamics; central‐peripheral GM‐CSF/CRP relationships	PTSD was associated with a smaller prefrontal‐limbic TSPO response, and greater suppression was associated with anhedonia	Small human sample; TSPO is not microglia‐specific, and the imaging and symptom relationships were associative	^181^
**B. Schizophrenia‐associated inflammatory and synapse‐handling programs**						
Schizophrenia	Postmortem dorsolateral prefrontal cortex stratified by inflammatory status; nine transcriptomic datasets across brain regions; postmortem prefrontal cortex from 62 matched case‐control pairs	Region‐ and subgroup‐dependent microglial and myeloid signatures	CD163/CD64/CCL2; microglial gene signatures; C4/C1q/AXL/MERTK/CD68	High‐inflammation cases showed stronger macrophage‐associated signals without uniform microglial activation; regional signatures and phagocytosis‐related genes differed across datasets	Cross‐sectional and partly bulk‐tissue evidence; cellular source and functional consequences could not be established	^187^, ^188^, ^189^
Schizophrenia	Mice transgenic for human C4A/C4B and C4A‐overexpressing mice with developmental dLGN and medial prefrontal cortex analyses	C4A‐dependent excessive microglial engulfment of synapses	C4A‐dependent complement tagging and microglial synaptic engulfment	C4A overexpression increased microglial synaptic engulfment, reduced cortical synapse density and altered social, anxiety‐like and spatial‐memory behaviours	Schizophrenia‐associated C4A risk was modelled in transgenic mice; direct human microglial functional validation was not performed	^190^
Schizophrenia	Patient‐derived monocyte‐induced microglia and iPSC‐neural cultures or synaptosomes; C4 genotyping and electronic health‐record analysis	Complement‐associated excessive synaptic uptake involving both microglial and synaptic abnormalities	Neuronal complement deposition and C4 risk variation; minocycline‐sensitive synapse uptake	Patient‐derived systems showed increased synapse engulfment, and minocycline reduced uptake in vitro	Induced microglia are not resident brain microglia; the electronic health‐record association did not establish preventive or treatment causality	^192^
Schizophrenia	Patient‐derived iPSC neurons and microglia‐like cells; forebrain organoids with microglia; developmental postmortem and genetic datasets	Kynurenic‐acid‐driven, activity‐dependent microglial synapse internalisation	Kynurenic acid; kynurenine aminotransferases; activity‐dependent synapse elimination	Kynurenic acid increased microglial uptake of synaptic structures, whereas inhibition of endogenous kynurenic‐acid production reduced synapse internalisation	Functional evidence came from patient‐derived cellular and organoid models; clinical efficacy of kynurenine‐aminotransferase inhibition was not tested	^193^
**C. Neurodevelopmental programs**						
ASD / SCN2A deficiency	Scn2a‐deficient mice; microglia‐incorporated human cerebral organoids carrying an ASD‐associated SCN2A protein‐truncating mutation	Developmental excessive microglial pruning of postsynaptic structures	SCN2A deficiency; complement C3‐associated pruning; PLX3397‐mediated microglial ablation	Excessive pruning accompanied reduced synaptic transmission and spine density; microglial ablation partially restored both, and mutant human organoids showed increased post‐synapse elimination	Behavioural and rescue evidence came from mice; human validation used cerebral organoids rather than in vivo brain	^199^
ASD / Moesin deficiency	Msn‐knockout mice with medial prefrontal cortex synaptic and behavioural analyses; IFNAR1‐antibody intervention	IFN‐associated, C1q‐dependent excessive microglial synaptic pruning	Msn–IFN/IFNAR1 signalling; C1q‐dependent pruning	Msn loss caused synaptic deficits, impaired social novelty and repetitive behaviour; IFNAR1 blockade rescued microglial abnormalities and social deficits	Global Msn knockout and systemic IFNAR1 blockade were not microglia‐specific; human functional validation was not reported	^201^
ASD / TMEM59 deficiency	Global and microglia‐specific Tmem59‐deficient mice; primary microglia‐neuron assays; human ASD expression datasets	Impaired microglial engulfment of excitatory synapses	TMEM59–CD93/C1q‐receptor stability; synaptic phagocytosis	TMEM59 deficiency reduced synapse engulfment, increased excitatory transmission and dendritic‐spine density and produced ASD‐like behaviours	Human support was based on peripheral or lymphoblastoid expression datasets; causal evidence was primarily from mouse models	^203^
ASD / TREM2 deficiency	Trem2‐knockout mice with synaptic, electrophysiological, connectivity and behavioural analyses; human autism TREM2‐protein association	TREM2‐dependent developmental microglial synapse elimination	TREM2‐mediated microglial synaptic refinement	Trem2 deficiency impaired synapse elimination, increased excitatory neurotransmission, reduced long‐range connectivity and produced repetitive behaviour and altered sociability	Causal evidence came from germline Trem2‐knockout mice; the human TREM2‐symptom relationship was correlational	^204^
Rett syndrome	Microglia‐specific Mecp2‐deficient mice and Mecp2‐null mice with genetic or pharmacological RIPK1 inhibition	Inflammatory, oxidative and glutamate‐release program	RIPK1; oxidative stress; SLC7A11/SLC38A1/GLS	RIPK1 inhibition reduced microglial inflammatory and glutamate‐release programs, ameliorated motor dysfunction and prolonged survival	Mouse evidence, predominantly using male Mecp2‐null models; relevance to the mainly female human disorder remains uncertain	^202^

## 7 DISCUSSION AND FUTURE PERSPECTIVES

New evidence suggests that microglial responses differ vastly depending on the stage of the disease, anatomical location in the brain and local microenvironment.^210^ Although inflammatory signalling, phagocytosis, metabolic regulation, lysosomal processing and intercellular communication are recurrent across the disease categories discussed here, their relative importance and functional consequences differ by context. Acute brain injury manifests as rapid spatiotemporal remodelling of inflammatory, metabolic and phagocytic programs while neurodegenerative and demyelinating disorders highlight pathological cargo handling, lysosomal processing and lipid metabolism.^211^ In contrast, for CNS infection there is also continued pathogen sensing (and clearance), antigen presentation and host defence; neurodevelopmental and psychiatric disorders have a more pronounced dependence on brain region and timing of commitment to a synaptic remodelling program. Thus, the state or change in inflammatory markers does not by itself determine whether a microglial response is beneficial or harmful,^212^ and common molecular pathways should not be assumed to represent interchangeable therapeutic targets across diseases. Functional interpretation requires taking into account the cellular process in which microglia are engaged, as well as the material and other cells encountered and the stage of disease.

Such microglial intervention may also be experienced quite differently depending on the conditions. Phagocytosis can aid in clearing damaged material, protein aggregates, myelin debris or pathogens; however, pathological and/or excessive phagocytic engulfment can enhance tissue injury. Such variability has been documented after microglial depletion and renewal in multiple experimental CNS disease and injury contexts, but the outcomes appear to depend on both pathological setting and the conditions of interventions; continuous depletion may also compromise essential functions of residual microglia and, overall, the efficiency as well as consequences of myeloid clearance and repopulation in humans remain largely uncharacterised.^213^ These results suggest that decreasing the activity or number of microglia cannot be assumed to yield a general therapeutic benefit. For each disease context, the relevant function and treatment window must be determined.

The above limitations help to explain the inconsistencies in findings across individual studies. Although a great deal of this causal evidence comes from rodent models, cultured microglia, human iPSC‐derived systems or short‐term experimental interventions, human brain studies are often confined to one or several regions and sampling stages. Studies comparing these cells directly have demonstrated that inflammatory stimulation can induce similar metabolic alterations in mouse and human microglia yet reveal intriguing differences in the regulation of the same pathways for these responses between species.^214^ The differentiation of resident microglia and infiltrating macrophages is another issue, given that their phenotypic characteristics may become increasingly similar during neuroinflammation.^215^ Finally, transcriptional states determined from single‐cell or spatial analyses do not confirm phagocytic capability, inflammatory output, tissue‐protective functions or behavioural benefit unless functionally validated. Additional factors, such as differences in age, sex, anatomical region, time of sampling, dose amount and timing of treatment, limit direct comparison between studies as well. These factors need to be considered when microglial markers are interpreted as positive therapeutic effects.

Such restrictions are also a hindrance to the translation of microglia‐targeted treatments. Broad suppression or depletion may abate harmful inflammatory activity but may also impair surveillance, pathogen defence, clearance and repair. Microglia replacement uses donor‐derived or genetically corrected cells, and although progress in translating this approach from experimental replacement to early human intervention has been made, further research is required with respect to long‐term function, safety,^216^ and engraftment efficiency. Other interventions that enhance delivery or modulate microglial responses may overcome some limitations of standard treatment, but these approaches also need scrutiny for their cellular specificity and duration of action. Evidence of clinical translation thus requires feasibility of delivery to the clinical target population, an appropriate temporal readout after disease initiation, followed by a durable and safe functional response.

Future studies should focus more on associating reproducible microglial states with distinct cellular functions, rather than merely continuing to add state classifications. More consistent reporting of anatomical region, disease stage, age, sex and sampling time would facilitate comparisons between studies, while integration of findings in human brains with CSF, blood biomarkers and imaging data may allow relevant microglial changes to be tracked longitudinally.^38^, ^217^ Downstream candidate targets should then be functionally validated in a cell‐type‐specific approach as well as characterised for delivery, treatment timing and long‐term safety. Near term, the field can evolve to complement therapeutic selection driven largely by transcriptomic state labels with perturbation‐associated frameworks that link spatially resolved microglial states to mechanistically defined cell functions and clinically pleiotropic biomarkers. Replacement of the endogenous microglial population could be biologically safer and feasible in primary microgliopathies, genetically defined disorders with direct correction of defective microglial development and function, although broader application will necessitate additional optimisation of disease‐stage‐specific efficacy, delivery, specificity and safety.^218^, ^219^ Ultimately, clinically actionable microglia‐directed therapy may be best selected not by diagnostic category alone, but rather by pairing the main cellular dysfunction with an appropriate biomarker, cell‐selective intervention and therapeutic window.

## AUTHOR CONTRIBUTIONS

Jie Chen, Wangzheqi Zhang and Lizhou Song contributed to the manuscript writing and figure preparation; Derong Cui, Yiyang Xia and Haoling Zhang designed the work; Yan Liao supervised the work. All the authors read and approved the final manuscript.

## CONFLICT OF INTEREST STATEMENT

The authors declare no conflicts of interest.

## ETHICS STATEMENT

Not applicable. This manuscript does not contain any studies with human participants or animals performed by any of the authors.

## CONSENT FOR PUBLICATION

Not applicable. This manuscript does not include details, images or videos relating to an individual person.

## Data Availability

Data sharing is not applicable to this article as no datasets were generated or directly analysed during the current study. All information is derived from published literature.
